# Review and Hypothesis: A Potential Common Link Between Glial Cells, Calcium Changes, Modulation of Synaptic Transmission, Spreading Depression, Migraine, and Epilepsy—H^+^

**DOI:** 10.3389/fncel.2021.693095

**Published:** 2021-09-03

**Authors:** Robert Paul Malchow, Boriana K. Tchernookova, Ji-in Vivien Choi, Peter J. S. Smith, Richard H. Kramer, Matthew A. Kreitzer

**Affiliations:** ^1^Department of Biological Sciences, University of Illinois at Chicago, Chicago, IL, United States; ^2^Department of Ophthalmology and Visual Sciences, University of Illinois at Chicago, Chicago, IL, United States; ^3^Stritch School of Medicine, Loyola University, Maywood, IL, United States; ^4^Institute for Life Sciences, University of Southampton, Highfield Campus, Southampton, United Kingdom; ^5^Bell Center, Marine Biological Laboratory, Woods Hole, MA, United States; ^6^Department of Molecular and Cell Biology, University of California, Berkeley, Berkeley, CA, United States; ^7^Department of Biology, Indiana Wesleyan University, Marion, IN, United States

**Keywords:** glia, Müller cell, pH, H^+^, ATP, epilepsy, migraine, spreading depression

## Abstract

There is significant evidence to support the notion that glial cells can modulate the strength of synaptic connections between nerve cells, and it has further been suggested that alterations in intracellular calcium are likely to play a key role in this process. However, the molecular mechanism(s) by which glial cells modulate neuronal signaling remains contentiously debated. Recent experiments have suggested that alterations in extracellular H^+^ efflux initiated by extracellular ATP may play a key role in the modulation of synaptic strength by radial glial cells in the retina and astrocytes throughout the brain. ATP-elicited alterations in H^+^ flux from radial glial cells were first detected from Müller cells enzymatically dissociated from the retina of tiger salamander using self-referencing H^+^-selective microelectrodes. The ATP-elicited alteration in H^+^ efflux was further found to be highly evolutionarily conserved, extending to Müller cells isolated from species as diverse as lamprey, skate, rat, mouse, monkey and human. More recently, self-referencing H^+^-selective electrodes have been used to detect ATP-elicited alterations in H^+^ efflux around individual mammalian astrocytes from the cortex and hippocampus. Tied to increases in intracellular calcium, these ATP-induced extracellular acidifications are well-positioned to be key mediators of synaptic modulation. In this article, we examine the evidence supporting H^+^ as a key modulator of neurotransmission, review data showing that extracellular ATP elicits an increase in H^+^ efflux from glial cells, and describe the potential signal transduction pathways involved in glial cell—mediated H^+^ efflux. We then examine the potential role that extracellular H^+^ released by glia might play in regulating synaptic transmission within the vertebrate retina, and then expand the focus to discuss potential roles in spreading depression, migraine, epilepsy, and alterations in brain rhythms, and suggest that alterations in extracellular H^+^ may be a unifying feature linking these disparate phenomena.

## Introduction

An ever-increasing number of studies suggest that cells christened by Rudolf Virchow as “glue”—glial cells—are more than the “passive” or “filler” elements originally envisaged years ago. In addition to providing nutrients and scaffolding critical for neuronal growth, proper development and continued function (see Barres et al., 2015; von Bernhardi, 2016), glia are now recognized as active participants in the “tripartite synapse,” modulating and regulating signal transmission between neurons and among themselves (Halassa et al., 2007, 2009; Papouin et al., 2017). It has long been suspected that elevations in glial intracellular calcium play a role in the modulation of synaptic transfer at synapses, but the nature and molecular mechanism(s) of such regulation is currently an area of contentious debate (see Khakh and McCarthy, 2015; Bazargani and Attwell, 2016; Guerra-Gomes et al., 2017; Fiacco and McCarthy, 2018; Savtchouk and Volterra, 2018; Ashhad and Narayanan, 2019; Semyanov et al., 2020; Kofuji and Araque, 2021). In addition, a number of “gliotransmitters” have been identified as potential modulators of neuronal activity, among them glutamate, ATP, serine, and GABA (Petrelli and Bezzi, 2016). The degree to which these contribute to the modulation of neurotransmitter release by neurons and the mechanisms regulating the release of these gliotransmitters remains controversial (Sahlender et al., 2014; Durkee and Araque, 2019).

A potent but commonly overlooked regulator of synaptic transmission is simple H^+^—that is, small changes in levels of extracellular acidity around sites of neurotransmitter release. Recent studies have shown that activation of glial cells inducing increased intracellular calcium also promotes the release of H^+^ from glia, and it has been proposed that this may play a key role in regulating synaptic transmission (Tchernookova et al., 2018, 2021; Choi et al., 2021). In this review and hypothesis article, we first review studies demonstrating the potency of extracellular H^+^ as a modulator of synaptic transmission and then describe techniques used and studies conducted to show calcium-dependent extrusion of H^+^ from glial cells activated by extracellular ATP. The potential implications of such glial-mediated H^+^ extrusion in the regulation of normal nervous system function as well as in such maladies as epilepsy and migraines are then discussed, followed by a perspective on challenges facing further advances in the field.

## H^+^—A Potent Modulator of Synaptic Transmission

Alterations in extracellular H^+^ can affect many channels and other proteins within the nervous system with consequent significant effects on its function. In this article, we restrict our examination to the effects of altered levels of extracellular H^+^ on mechanisms underlying synaptic transmission.

**Figure 1:**
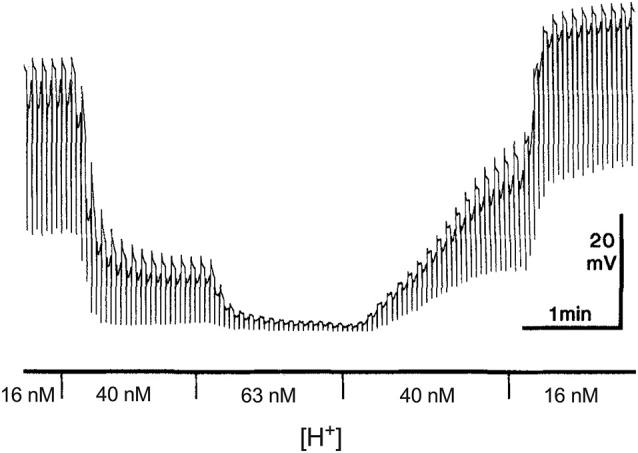
Dependence of the dark resting potential and light-evoked responses of a retinal horizontal cell of the salamander to alterations in extracellular H^+^. Modified from Kleinschmidt (1991). An intracellular sharp electrode was used to monitor membrane potential in a single horizontal cell impaled in an isolated retinal preparation superfused with the pH of the solution buffered with 20 mM HEPES. Alternating red (600 nm) and green (555 nm) 100 ms full field flashes, separated by 3 s and adjusted to give equal cone responses were used to stimulate the cell. The author notes that the responses to the different lights can be distinguished by the small rod-driven tails found only in responses to green stimuli.

Just how potent small alterations in extracellular H^+^ are in affecting neurotransmitter release is perhaps best illustrated in Figure 1, a modification of a figure first published by Kleinschmidt (1991). This figure shows electrical responses from a neuron in the retina of a salamander made using a high resistance intracellular pipette. The cell recorded is a horizontal cell that receives direct input from photoreceptors and whose primary response to a flash of light is a hyperpolarization, shown as a downward deflection in the figure. In the dark, vertebrate photoreceptors sit depolarized and are thought to continuously release glutamate onto postsynaptic neurons (for review, see Wu, 1994; Barnes, 1995). Glutamate binds to ionotropic channels on horizontal cells, allowing sodium and calcium influx, and horizontal cells thus sit relatively depolarized in the dark. Absorption of light induces a hyperpolarization of photoreceptors, which closes voltage-gated calcium channels present on photoreceptor axon terminals. The reduction in calcium influx decreases the calcium-dependent release of glutamate from photoreceptors. The glutamate-sensitive channels on horizontal cells close when extracellular glutamate is not present, inducing a hyperpolarization of the horizontal cell. The figure shows the multiple downward hyperpolarizations in a horizontal cell resulting from repetitive bright light stimuli.

The first responses shown in the figure were obtained with the isolated retina superfused with a Ringer’s solution containing 16 nM H^+^ using the pH buffer HEPES. In this condition, the cell sits at a depolarized level and light-induced hyperpolarizations are large. The solution was then switched to one containing 40 nM H^+^, and there are two clear effects of this modest increase in extracellular H^+^ concentration. First, a large hyperpolarization of the cell is observed, and second, a significant decrease occurs in hyperpolarizations induced by light. A third change to a solution containing 63 nM H^+^ results in a further hyperpolarization of the cell and the virtual elimination of hyperpolarizations induced by light. The effects of altered levels of H^+^ on the baseline level of polarization and hyperpolarizations induced by light were fully and rapidly reversible, as the second portion of the recording makes clear.

To emphasize how sensitive both the resting membrane potential and responses to light are to levels of extracellular H^+^, the concentration of H^+^ in the solutions used—16 nM, 40 nM and 63 nM—have been converted from the equivalent levels of pH listed in the original figure (pH_o_ values of 7.8, 7.4 and 7.2, respectively). But it is not only the low concentration of H^+^ relative to that of other neurotransmitters/neuromodulators that is worth noting; Kleinschmidt also emphasized how extraordinarily steep the relation between altered levels of extracellular H^+^ and resting membrane potential is, estimating a Hill coefficient of about 4 with an inflection point of 23 nM (pH = 7.64). The sensitivity of horizontal cells to direct application of glutamate was unchanged by these alterations in extracellular H^+^, implicating a presynaptic localization for the effect, and light-induced hyperpolarizations of photoreceptors were only weakly altered, leading to a conclusion that the dramatic effects of altered H^+^ resulted from inhibition in the release of neurotransmitter from photoreceptors to second order cells. The steep dependence of horizontal cell membrane potential and light-induced responses on alterations of extracellular H^+^ has been noted by others; for example, in recordings from horizontal cells of goldfish, increasing the H^+^ concentration of a solution superfusing the retina from 25 nM to 40 nM, which reduced the value of extracellular pH by 0.1 unit within the retina as measured by H^+^-selective microelectrodes, reduced cone horizontal cell light responses by about 50% (Harsanyi and Mangel, 1993; Dmitriev and Mangel, 2000; Mangel, 2001).

Barnes et al. (1993) provided data that the effect of altered levels of H^+^ occurred largely at voltage-gated calcium channels of photoreceptors. The exponential dependence on extracellular H^+^ of both L-type calcium channel currents and light-induced responses of second order neurons was characterized, and the authors noted that changing the extracellular H^+^ concentration to 115 nM, close to neutral (pH = 6.94), was as effective in blocking calcium influx through voltage-gated calcium channels and responses from second order neurons as was the addition of 100 μM cadmium, which blocked virtually all synaptic transmission. Increased levels of extracellular H^+^ reduced the maximal current through calcium channels and shifted the activation voltage needed to open the channels to significantly more positive transmembrane potentials. In examining the molecular basis of the block of L-type calcium channels by H^+^, Chen et al. (1996) demonstrated that protons exerted their action on calcium channels by binding to four glutamate residues located within the channel pore. They further noted that because of the high degree of conservation of the glutamate residues in the pore region of all voltage-gated calcium channels, the pronounced sensitivity to extracellular H^+^ was likely to be a universal characteristic of this family of membrane proteins. Confirmation of this prediction came from work by Doering and McRory (2007) examining the effects of extracellular H^+^ on nine different subtypes of voltage gated calcium channels, observing that in all cases, acidification of the extracellular solution resulted in a significant depolarizing shift in the activation curve and reduction in peak current amplitudes. Thus, increases in H^+^ around the physiological range are likely to reduce the influx of calcium into neuronal terminals and decrease significantly calcium-dependent neurotransmitter release at virtually all chemical synapses within the nervous system.

Alterations in extracellular H^+^ also have potent effects at several other steps in the process of synaptic transmission. For example, although the AMPA subtype of glutamate receptors present on horizontal cells examined by Kleinschmidt and Barnes were not particularly sensitive to physiologically relevant changes in extracellular H^+^, NMDA glutamate receptors are highly sensitive to small extracellular acidifications around the physiological range (Traynelis and Cull-Candy, 1990; Traynelis et al., 1995, 2010; Jalali-Yazdi et al., 2018). These receptors, believed to be critically important in learning and memory and expressed widely in neurons throughout the nervous system, have an IC_50_ for inhibition by H^+^ of about 50 nM (pH of 7.3), a value close to normal levels of extracellular interstitial pH of many organisms. Indeed, the size of postsynaptic NMDA receptor currents of hippocampal pyramidal neurons was boosted by approximately one third through extracellular alkalinization elicited by activation of the same cell’s PMCA calcium pump, which extrudes one calcium while transporting two protons into the cell during each cycle of transport (Chen and Chesler, 2015).

Additionally, the reuptake of neurotransmitters from the extracellular space into cells adjacent to release sites significantly influences the overall impact of neurotransmitters released during synaptic transmission. Removal of neurotransmitters from the extracellular space surrounding the release site often requires co-transport with extracellular H^+^. For example, hippocampal astrocytes possess transport proteins for glutamate that ferry three sodium ions and one proton along with glutamate into the cell while also exporting one potassium ion (Nicholls and Attwell, 1990; Owe et al., 2006; Vandenberg and Ryan, 2013; Rose et al., 2017). In an examination of flux coupling of the EAAT3 glutamate transporter, Zerangue and Kavanaugh (1996) reported an affinity constant for H^+^ of 26 nM during glutamate transport, corresponding to an extracellular pH of 7.58. This value implies that small changes of extracellular H^+^ around normal physiological levels could significantly impact the transport of glutamate and thus alter the extent of effects induced by neurotransmitters on post-synaptic cells. The removal of glutamate by uptake into astrocytes leads to an extracellular alkalinization, and if extracellular levels of H^+^ remained depressed, the remaining glutamate could potentially linger longer and exert more pronounced effects.

Thus, several critical components of the process of synaptic transmission—the initial calcium-dependent release of neurotransmitters, its effects on certain post-synaptic receptors, and removal of neurotransmitters from the synaptic cleft—are all susceptible to small changes in extracellular H^+^ concentrations around the physiological range.

## H^+^ Efflux from Glial Cells Elicited by ATP

Recent studies using self-referencing H^+^-selective electrodes have demonstrated clear rises in extracellular H^+^ adjacent to radial glial cells isolated from the vertebrate retina and from astrocytes cultured from mice and rat hippocampus and cortex upon activation by extracellular ATP. In this section, we first describe the method used to detect H^+^ fluxes from individual cells, then show the nature of responses and finally describe what is known about the signal transduction process underlying the efflux of H^+^ from glia.

Attempts to definitively ascribe changes in extracellular levels of H^+^ to a particular cell type such as glia in the nervous system, with its extraordinarily complex web of processes, cell types, and panoply of H^+^-coupled transport proteins and channels, are exceptionally difficult. To ensure that measured changes in extracellular H^+^ come specifically from glial cells, therefore, initial characterizations are best carried out in cell culture preparations where responses can be obtained from cells unambiguously identified as glia and which sit isolated from neurons or other cell types present in the culture. While this approach ensures that measured signals emanate solely from cells identified as glia, the disruption of the normal extracellular architecture presents its own new technical hurdle: protons released by cells no longer can accumulate in the very small extracellular spaces and microdomains that allow small alterations in H^+^ activity to result in large overall changes in concentration in a small volume. A major challenge in measuring changes in extracellular H^+^ induced by activation of single isolated glial cells is that protons released by glia under such conditions diffuse quickly into the vast ocean of fluid surrounding the cells, making it quite difficult to measure changes in extracellular H^+^. Self-referencing H^+^-selective microelectrodes provide the needed spatial and temporal sensitivity and stability over time to make such measurements. A detailed description of the construction, use, advantages, and limitations of self-referencing electrodes is beyond the scope of this article; detailed reviews can be found in Smith (1995), Smith et al. (1999), Smith and Trimarchi (2001), Messerli et al. (2006), Smith et al. (2007), and Messerli and Smith (2010). Here, we provide a brief overview of the technique, highlighting how it works, why it greatly increases the useful sensitivity of H^+^-selective microelectrodes, and describe concerns and limitations to be kept in mind when using the technique.

**Figure 2:**
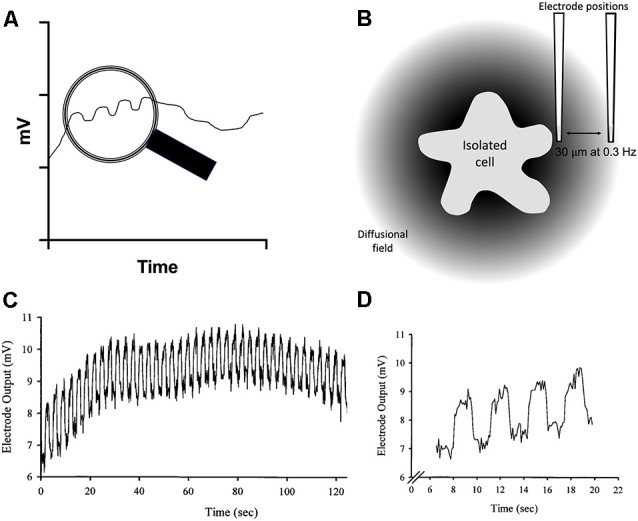
Schematic diagram and sample data illustrating the challenge imposed in using liquid cocktail-based ion-selective microelectrodes to measure changes in extracellular ion levels adjacent to cells, and the solution provided by using the electrode in a self-referencing fashion. **(A)** Drawing showing the electrical drift typical of such ion-selective electrodes as a function of time. The voltage output signal can wander several millivolts over several minutes. The view within the magnifying glass shows the small voltages one would expect to obtain by monitoring the electrode output at two positions, one close to the source of the ion, and the other a defined distance away from the cell. **(B)** Illustration of the diffusional field of an ion emanating from a cell and its decline with distance, along with the positioning of a self-referencing ion-selective electrode at the near pole, close to the source of ion efflux from the cell, and again at a position 30 μm away from the cell. **(C)** Experimental demonstration of electrical voltage drift from an ion-selective electrode; from Smith et al. (1999). The trace shows the output from a calcium-selective microelectrode while recording the response at both the near- and far-pole position from a source pipette filled with calcium. Note that the electrical drift is in the millivolt range; in biological preparations, differential voltages induced by physiological stimuli would be expected to be in the microvolt range. **(D)** An expanded section from **(C)** to more clearly illustrate the signal and the drift. Note how the electrical drift is common to both measuring positions in the electrical output signal.

In experiments using self-referencing electrodes to measure H^+^ fluxes from isolated glial cells, silanized glass pipettes with dimensions similar to patch pipettes are filled with an ~30 μm column of a liquid cocktail possessing high selectivity for H^+^ ions. The tips of the pipettes are then positioned 1–2 μm from the membrane of a cell. Ion-selective potentiometric electrodes report a voltage at the tip that varies as H^+^ ion activity changes as defined by the Nernst equation—a 10-fold change in concentration (or more strictly, activity) of H^+^ induces a 58 mV change at standard room temperatures. A major challenge encountered with the use of liquid cocktail-based ion-selective electrodes is random electrical slow-frequency baseline drift of the signal with time, drift more than large enough to swamp H^+^-generated signals expected from single isolated cells. The problem is schematically illustrated in Figure 2, along with the solution afforded by using the electrodes in a self-referencing configuration. A key facet of the electrical drift in the output signal is that while it is random, it is relatively slow, and is present regardless of whether the electrode is adjacent to a cell or a location far away. If the sensor is translated quickly enough—from a point adjacent to a cell to a second point away from the cell (in our experiments, 30 μm), the voltage contributed by drift will be essentially the same, or common, to the two points. External membrane-associated voltage gradients are too small to be detected by the H^+^-selective electrode, a very different situation from operating ion-selective electrodes in an intracellular environment. The possible but unlikely impact of surface charge influencing measurements is discussed in detail in Smith et al. (2010). An example of the actual size and time-dependence of electrical drift associated with ion-selective electrodes is shown in Figures 2C,D (note that this signal was generated by creating a steep artificial gradient from a source pipette far larger than would be expected when recording responses from a single cell, which generate signals in the microvolt range). Subtracting the output signal at the location distant from the cell (the far pole) from the voltage measured close to the cell (near pole) then minimizes the signal contributed by the slow electrical drift or common interferents. The subtraction results in a differential voltage that reflects the difference in H^+^ ion activity measured between the two points—that is, close to the cell compared to the value obtained 30 μm away, with a greatly reduced contribution associated with electrical drift. This simple process enhances the detection limits of liquid cocktail-based ion-selective electrodes by more than 1,000× (Somieski and Nagel, 2001), and enables the stable measurement of H^+^ fluxes from individual isolated cells (Smith and Trimarchi, 2001). It is worth noting that attempts to use two separate electrodes at the two positions will not work in this framework, since the electrical drift present in the two electrodes will vary independently and so cannot be subtracted out meaningfully to yield a stable baseline.

The enhanced sensitivity provided by self-referencing comes with its own challenges and limitations. First, the method is relatively slow. Electrodes are typically translated in an approximate square wave between the poles with an interval of 0.3 Hz, with data collected at each position for around 1 s. The electrode must be moved quickly enough so that the contribution from electrical drift is roughly the same at both locations, but not so quickly as to significantly stir the solution and thus potentially disrupt the differential H^+^ gradient at the heart of the measurement. With these operating parameters and dimensions, and taking into account the diffusion constant of H^+^, mixing is not seen as a problem (Messerli et al., 2006; Smith et al., 2010).

Second, the differential flux signal depends critically on the distance of the electrode from the source, with the signal from a point source declining exponentially due to diffusion. The electrode must be placed close enough to an H^+^ source to measure a differential signal, but not so close as to touch the membrane of the cell, which would result in the silanized glass and liquid cocktail disrupting the plasma membrane. Thus, the absolute magnitude of the differential signal will vary significantly with the distance of the electrode away from the cell and must be kept constant.

Third, as the electrode tip surface area is exposed to the external medium, pharmacological agents added to the medium could affect the performance of the H^+^-selective liquid cocktail. It is essential to conduct control experiments to ensure that the addition of a drug at an experimental concentration, does not alter the selectivity or sensitivity of the electrodes. Another technical matter when applying pharmacological modulators is the instability of the baseline during application, which tends to rule out continuous superfusion. Modulators are either applied by bulk replacement of the medium or as aliquots at a distance from the cell.

Fourth, perhaps the most significant modulator of recorded H^+^ flux is the pH buffer in the solution. Messerli et al. (2006) deal with this in-depth and discuss how the proportion of protonated vs. unprotonated buffer can be taken into account. Frequently, however, results are presented in a qualitative manner without attempting conversion to a flux value.

Finally, self-referencing H^+^-selective electrodes report an ion flux—that is, an alteration in H^+^-activity as it changes over a specified period of time and distance, typically given in units of μmol cm^−2^^−1^. As noted above, an exact determination of the flux value is dependent on distance, response times, buffering capacity, the source strength, and *in vivo*, on the intracellular volume and tortuosity (Syková and Nicholson, 2008; Smith et al., 2010). Given these considerations, we have opted to present results simply as the difference in the voltage reported between the two poles of translation, i.e., Δ μV.

**Figure 3:**
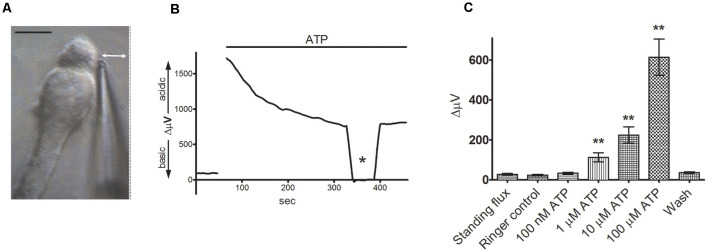
Extracellular ATP induces a significant increase in extracellular H^+^ flux from isolated Müller cells. From Tchernookova et al. (2018). **(A)** An example of an isolated Müller cell with a self-referencing H^+^-selective microelectrode positioned next to the apical end of the cell. Scale bar: 20 μm; double-headed arrow depicts the direction of electrode movement as it alternately records the potential established by protons adjacent to the cell and 30 μm away. **(B)** Response from a single isolated Müller cell to 100 μM ATP. The top bar represents the duration of drug application. Asterisk represents a background control reading taken 200 μm above the cell. **(C)** Mean data from eight trials in response to various extracellular ATP concentrations: error bars represent standard errors of the mean. The double asterisks indicate statistical *P*-values of 0.01 or less.

The application of self-referencing H^+^-selective electrodes to detect H^+^ fluxes from isolated retinal glial cells is shown in Figure 3. To obtain isolated glia, retinae from tiger salamanders were dissociated using a commonly used and well-characterized papain-based enzymatic dissociation protocol. The radial glia referred to as Müller cells, possess a distinct morphology that allows ready and precise identification, with cells possessing an apical tuft and a thick stalk connecting cell body and basal end foot regions, along with fine processes extruding from the plasma membrane (see Newman, 1985). Figure 3A shows a self-referencing H^+^ sensor placed adjacent to the junction of the apical tuft and cell body region of the cell, a location likely to correspond with what would be the outer plexiform layer (OPL) of the intact retina. Figure 3B shows a typical recording obtained from a single Müller cell using an H^+^-selective microelectrode (Tchernookova et al., 2018). A small initial standing H^+^ flux is first observed, indicating that the reading obtained adjacent to the cell is more acidic than the point 30 μm away, revealing a small basal efflux of H^+^. A bolus of ATP resulting in a final concentration of 100 μM induced a large increase in H^+^ flux from the cell. At the point marked with an asterisk, the electrode was moved to a control location 200 μm above the cell. At this location, the concentration of H^+^ should be roughly identical at the two electrode positions, and consequently, the differential voltage should be close to zero; this is an important control incorporated in virtually every experiment to ensure the self-referencing system is operating correctly. Returning the self-referencing H^+^-selective electrode to its position adjacent to the cell results in a restoration of the H^+^ flux differential signal. Figure 3C shows the dose-response relation of H^+^ flux to changes in extracellular ATP, and it is notable that concentrations of extracellular ATP as low as 1 μM initiate significant H^+^ efflux. At high doses of extracellular ATP, the size of the H^+^ flux signal was on the same order of magnitude as that detected from cells and tissues known to be potent proton pumpers, such as in the vas deferens, where changes in extracellular H^+^ concentration play an essential role in the activation of sperm cells (Breton et al., 1998). The extracellular H^+^ fluxes induced by ATP from Müller cells were mimicked by the P2Y1 agonist MRS 2365 and were significantly reduced by the P2 receptor blockers suramin and PPADS, suggesting activation of P2Y receptors. ADP, UTP, and the non-hydrolyzable analog ATPγs were also potent stimulators of extracellular H^+^ fluxes, but 100 μM adenosine had no effect.

The alterations in H^+^ flux induced by extracellular ATP did not appear to result from sodium-coupled bicarbonate transporters. The experiments described above were carried out in solutions in which the primary extracellular H^+^ buffer was 1 mM HEPES, with no bicarbonate added. ATP-elicited increases in H^+^ flux from Müller cells also persisted unchanged when solutions were bubbled for 15 min before use with 100% oxygen, to remove trace amounts of CO_2_ from the atmosphere that could potentially be used by very high affinity bicarbonate transporters (Theparambil et al., 2014; Tchernookova et al., 2021).

**Figure 4:**
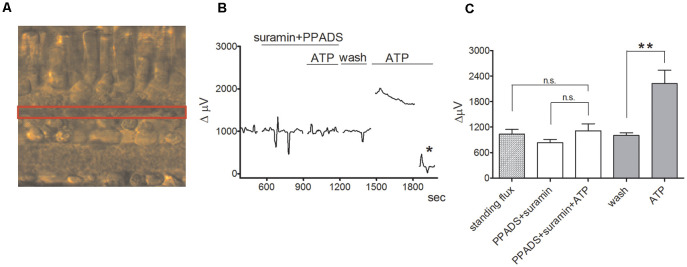
ATP induces an increase in extracellular H^+^ flux at the outer plexiform layer (OPL) in retinal slices that is markedly reduced by PPADS and suramin. From Tchernookova et al. (2018). **(A)** Retinal slice preparation obtained using a 200 μm section of a tiger salamander retina. The red box highlights the outer plexiform (synaptic) layer (OPL). **(B)** A representative trace showing the response observed to the application of 100 μM ATP from an individual self-referencing recording from a retinal slice with the microelectrode positioned just above the OPL; asterisk indicates a background control reading taken 600 μm above the retinal slice.** (C)** Mean data from eight slices. ATP induced a significantly smaller increase in extracellular H^+^ flux in the background of suramin and PPADS than it did in plain Ringer’s solution. The double asterisks indicate statistical *P*-values of 0.01 or less. n.s., not significantly different.

Significant increases in extracellular H^+^ flux induced by extracellular ATP were also detected when the normal complex web of cellular connections within the retina was preserved in retinal slice preparations. Figure 4 shows results with a self-referencing H^+^-selective electrode placed over the outer plexiform (synaptic) layer of a tiger salamander retinal slice and then alternately translated to a position 30 μm above the retinal slice to obtain a differential signal (Figure 4A). The application of 100 μM extracellular ATP gave a robust increase in H^+^ flux that was substantially reduced by the P2 receptor inhibitors PPADS and suramin (Figures 4B,C). Similar increases in extracellular H^+^ flux induced by ATP were observed when the H^+^-selective microelectrode was placed above the inner plexiform (synaptic) layer of the retina and were also reduced by application of PPADS and suramin. Recordings from isolated Müller cells made adjacent to the side of the basal end-foot of cells revealed extracellular ATP-induced increases in H^+^ flux as well, consistent with increased H^+^ flux detected at the inner synaptic layer of the retina.

**Figure 5:**
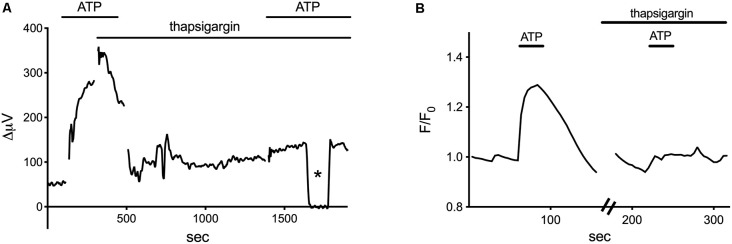
Thapsigargin, an inhibitor of calcium reuptake into intracellular stores, blocks both the ATP-elicited increase in H^+^ and the ATP-elicited increase in calcium from isolated Müller cells. Modified from Tchernookova et al. (2018). **(A)** A representative self-referencing recording from an isolated Müller cell demonstrating an initial increase in H^+^ flux induced by 1 μM ATP; after soaking the cell for about 15 min with 1 μM thapsigargin, the ATP-induced alteration in H^+^ flux was largely blocked. Asterisk indicates a control reading taken 200 μm from the cell. **(B)** Changes in intracellular calcium induced by ATP as reported by alterations in fluorescence of the calcium indicator Oregon Green. 1 μM ATP produced a robust increase in intracellular calcium. Subsequent ATP-induced alterations in Oregon Green fluorescence were blocked by soaking the cell in 1 μM thapsigargin for 15 min.

ATP-elicited increases in H^+^ flux from retinal radial glial cells appear to be mediated by activation of a G-protein cascade that induces an increase in calcium release from intracellular stores. Inhibition of the G-protein-coupled PLC linked to metabotropic ATP receptors by U-73122 reduced both the amplitude of ATP-elicited H^+^ efflux from isolated Müller cells as well as increases in intracellular calcium so commonly elicited in many glial cells by application of ATP. Both ATP-elicited increases in H^+^ efflux from Müller cells and concomitant increases in intracellular calcium were also reduced by the IP3 receptor inhibitor 2-APB. Finally, as shown in Figure 5, thapsigargin, an agent which depletes intracellular calcium by preventing reuptake into intracellular stores, potently reduced both the ATP-elicited increase in intracellular calcium (Figure 5A) as well as the ATP-initiated increase in H^+^ flux (Figure 5B). These data demonstrate that ATP-mediated increases in H^+^ flux from Müller cells are critically dependent upon increases in intracellular calcium released from intracellular stores.

**Figure 6:**
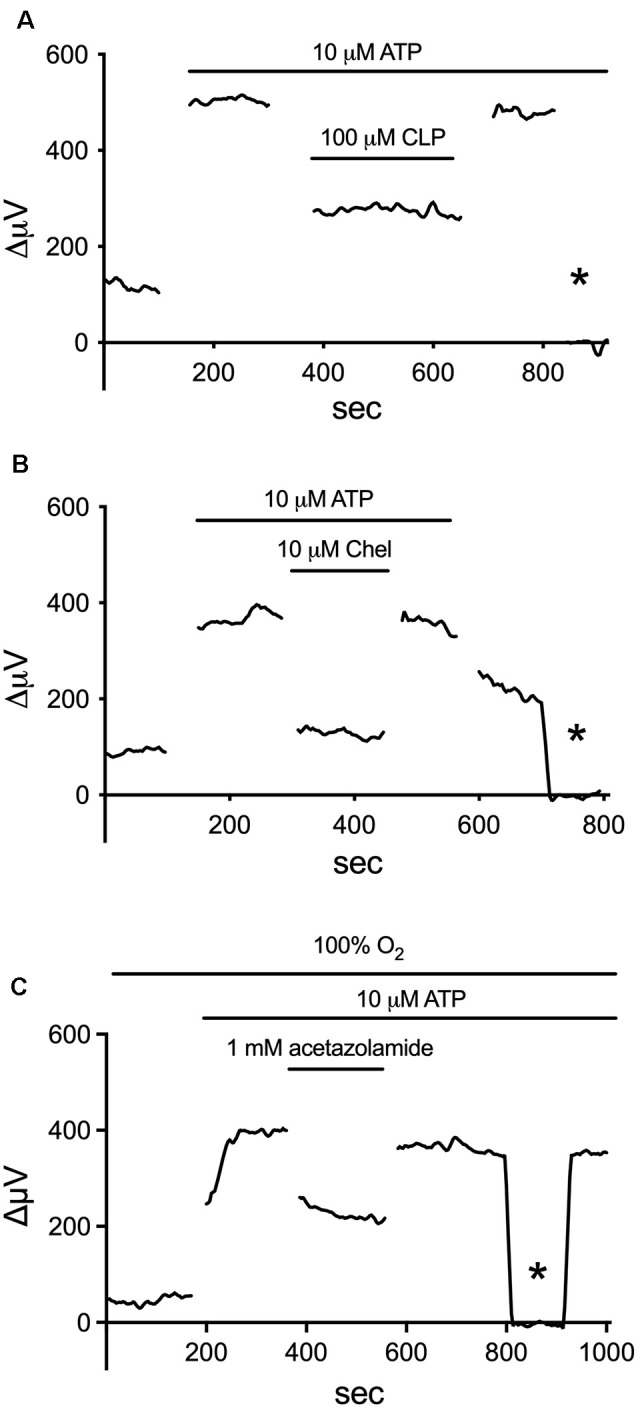
Inhibitors of calmodulin, PKC, and carbonic anhydrase (CA) reduce the ATP-elicited increase in H^+^ flux from retinal Müller cells isolated from the retina of the tiger salamander. **(A)** 100 μM of the calmodulin inhibitor CLP potently inhibited the rise in H^+^ flux from an isolated Müller cell induced by 10 μM ATP. **(B)** Reduction of ATP-elicited increase in H^+^ flux from a single tiger salamander Müller cell induced by 10 μM ATP upon the addition of 10 μM of the PKC inhibitor chelerythrine. **(C)** Inhibition of H^+^ efflux from a single Müller cell by 1 mM of the carbonic anhydrase inhibitor acetazolamide. Asterisk represents a background control reading taken 200 μm above the cell.

Additional experiments demonstrated that about 75% of the ATP-elicited increase in H^+^ flux likely resulted from the extrusion of protons *via* Na^+^/H^+^ exchange (Tchernookova et al., 2021). The complete removal of extracellular sodium coupled with replacement by choline reduced ATP-elicited increases in H^+^ efflux by 75%, and ATP-initiated H^+^ efflux was also significantly reduced by pharmacological agents known to inhibit Na^+^/H^+^ exchange, including amiloride, cariporide, and zoniporide. Additional unpublished data implicate activation of calmodulin and PKC in this process, examples of which are provided in Figure 6. The calmodulin inhibitors W-7 and chlorpromazine (CLP) at 100 μM and trifluoperazine (TFP) at 50 μM were all potent inhibitors of the ATP-initiated increase in H^+^ flux from tiger salamander Müller cells. The response from one cell to the application of ATP in the presence of a calmodulin inhibitor is shown in Figure 6A; in eight cells tested, the total H^+^ flux from isolated tiger salamander Müller cells by 10 μM ATP was 36 ± 12 μV in 100 μM CLP compared to a value of 239 ± 46 μV obtained from six separate cells in response to 10 μM ATP without CLP present. The block of H^+^ flux by these calmodulin inhibitors occurred without any noticeable effect on ATP-induced rises of intracellular calcium reported by the calcium indicator Oregon Green. The increase in H^+^ efflux from isolated tiger salamander Müller cells in response to 10 μM ATP was also significantly reduced by 10 μM of the PKC inhibitor chelerythrine, as shown in Figure 6B, which charts the H^+^ flux signal from a single cell first to 10 μM ATP, then with ATP in the presence of chelerythrine, and then with chelerythrine removed but ATP still present. A similar reduction of H^+^ efflux in response to ATP was observed in seven cells. The block of the ATP-elicited increase in H^+^ by chelerythrine also occurred without significant alteration in ATP-elicited increases in intracellular calcium as measured by Oregon Green fluorescence, suggesting a direct effect of chelerythrine on PKC. These data suggest that one important consequence of increased intracellular calcium initiated by ATP in retinal radial glia may be to activate calmodulin and PKC, which then promote an increase in Na^+^/H^+^ exchange activity.

**Figure 7:**
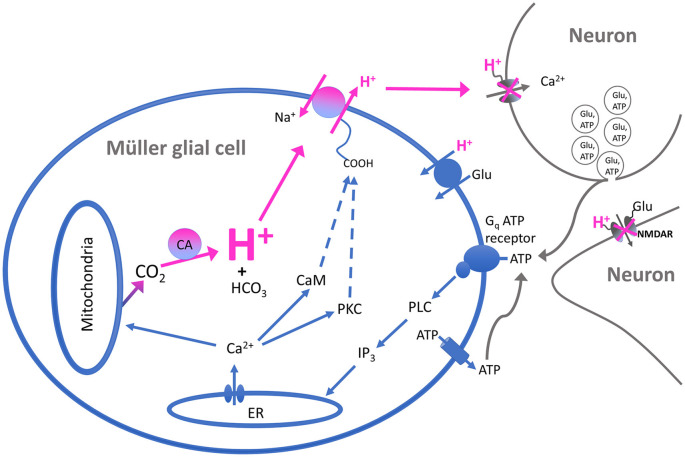
Schematic of the proposed signal transduction pathway underlying ATP-elicited H^+^ efflux from Müller cells of the tiger salamander. Extracellular ATP binds to a G-protein-coupled P2 receptor (labeled Gq ATP receptor) and activates the enzyme phospholipase C (PLC). This results in the production of inositol triphosphate (IP3) which promotes the release of calcium from intracellular stores such as the endoplasmic reticulum (ER). The rise in intracellular calcium leads to activation of calmodulin and protein kinase C (PKC), which in turn stimulate the activity of Na^+^/H^+^ exchange and the extrusion of H^+^ into the extracellular space. The rise in intracellular calcium is also postulated to stimulate mitochondria to produce a cloud of CO_2_ within the cell, serving as the substrate for the production of H^+^ and HCO_3_ by CA within the cell and providing continued high levels of intracellular H^+^ for export by transporters in the plasma membrane. The exported H^+^ ions act to bind to and inhibit voltage-gated calcium channels regulating neurotransmitter release from presynaptic neurons, as well as binding to and inhibiting NMDA-glutamate receptors on post-synaptic cells. The increased concentration of extracellular H^+^ is also postulated to facilitate the removal of neurotransmitters such as glutamate by H^+^-dependent transport proteins present on neurons and glia. The source of the extracellular ATP as shown in the model is proposed to come from co-release with neurotransmitters upon fusion of synaptic vesicles with the plasma membranes of presynaptic neurons; extracellular ATP can also arise from release by the glia themselves.

The H^+^ flux initiated by extracellular ATP from Müller cells is also significantly reduced by compounds that inhibit the activity of carbonic anhydrase (CA). Figure 6C shows the effect of one such compound, acetazolamide, on ATP-initiated H^+^ efflux. Previous studies have shown that methazolamide and acetazolamide significantly inhibit the activity of intracellular CA of isolated Müller cells (Newman, 1994). CAs play a key role in the production of H^+^ from the interaction of CO_2_ and water, increasing the speed of the chemical reaction by as much as a million times from its basal rate (Maren, 1988). Many glial cells, including the Müller cells of the retina, have high concentrations of CA intracellularly (see Cammer and Tansey, 1988; Nagelhus, 2005; Theparambil et al., 2017, 2020). The ability of membrane-permeant CA inhibitors to reduce H^+^ efflux initiated by extracellular ATP suggests that the rise in intracellular calcium induced by extracellular ATP facilitates the conversion of water and CO_2_ to H^+^ and HCO_3_. We hypothesize that the rise of intracellular calcium activates mitochondria and enhances metabolic activity within the cell (for review, see Boyman et al., 2020), leading to the production of CO_2_, effectively providing an increase in the substrate for the reaction facilitated by CA. This would result in increases in intracellular levels of H^+^, which would then be exported from the cell largely *via* Na^+^/H^+^ exchange. A schematic diagram illustrating our current thoughts regarding the signal transduction process leading to the increase in H^+^ efflux initiated by extracellular ATP is shown in Figure 7.

Increases in extracellular H^+^ efflux in response to small increases in extracellular ATP appear to be a common and highly conserved response detectable in Müller cells isolated from a wide range of other organisms. In addition to Müller cells of the tiger salamander, self-referencing H^+^-selective recordings from Müller cells isolated from such distantly related animals as lamprey, skate, catfish, rat, two species of monkey, as well as from human donor retinal tissue, all showed significant increases in H^+^ efflux by 100 μM ATP (Tchernookova et al., 2018). The high degree of conservation of ATP-elicited increase in H^+^ efflux across such disparate species speaks of a highly important biological function conserved across organisms.

**Figure 8:**
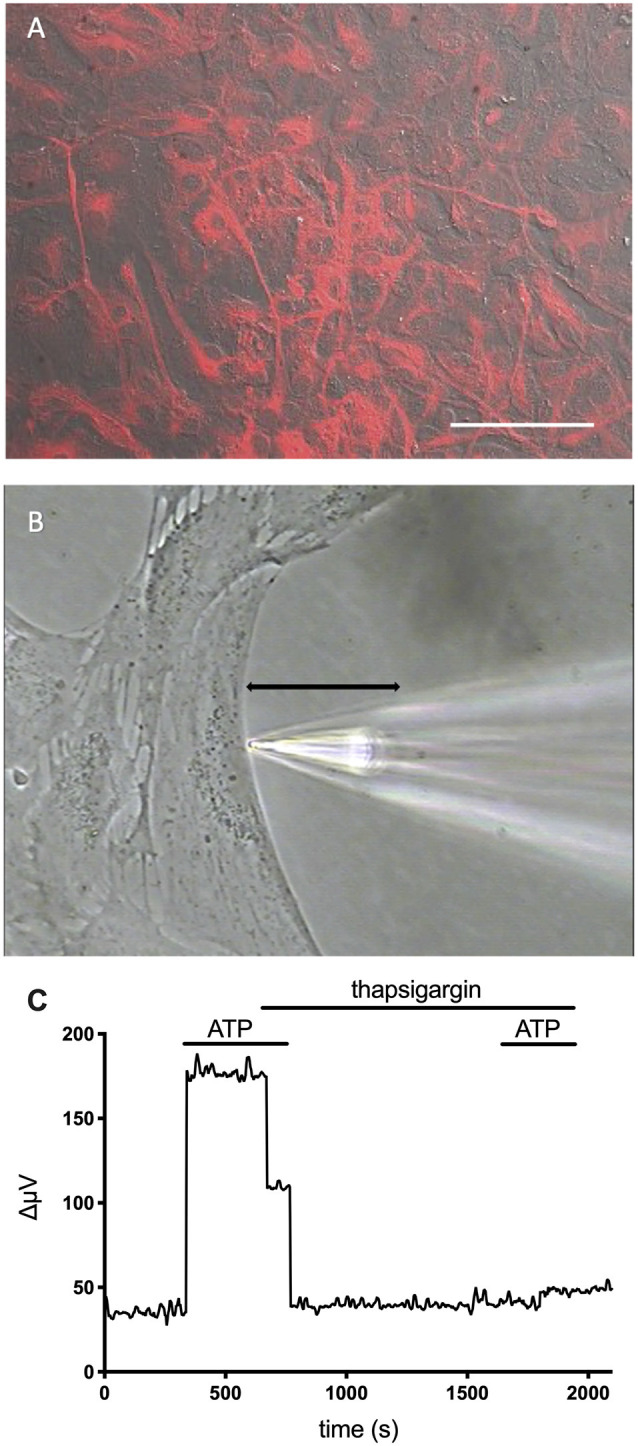
Cells cultured from the hippocampus of mice and identified as astrocytes show ATP-mediated increases in H^+^ efflux that are greatly reduced by the calcium reuptake blocker thapsigargin. Modified from Choi et al. (2021). **(A)** Low power photomicrograph combining DIC image with fluorescent staining for glial fibrillary acidic protein (GFAP) in a confluent dish of cells cultured for 11 days from mouse hippocampus. Red indicates signal from GFAP. The signal was not observed in negative controls (not shown). Scale bar = 100 μm. **(B)** High power photomicrograph showing the positioning of an H^+^ selective microelectrode adjacent to a cell cultured from mouse hippocampus. The black line indicates the 30 μm lateral movement the H^+^ selective electrode would travel from the near pole adjacent to the plasma membrane (pictured) to a background location 30 μm away. **(C)** H^+^ efflux from a single hippocampal astrocyte induced by 100 μM ATP first under normal conditions and then after soaking the cell in 1 μM thapsigargin. As is apparent, thapsigargin virtually abolished the increase in H^+^ flux initiated by ATP. In control cells, responses to a second application of ATP in plain Ringer’s solution without thapsigargin present were not significantly different from initial responses to ATP (data not shown).

Extracellular ATP also elicits increases in extracellular H^+^ fluxes from astrocytes cultured from mouse hippocampus and rat cortex (Choi et al., 2021). Cultured cells were identified as astrocytes by high levels of the intermediate filament protein glial fibrillary acidic protein (GFAP). Figure 8A shows the high level of GFAP staining typically observed in such cultures using an antibody specific to GFAP, and Figure 8B shows the placement of an H^+^-selective electrode adjacent to a cell identified as an astrocyte. The ATP-elicited increase in H^+^ efflux from cultured astrocytes appeared to be independent of bicarbonate transport activity since ATP increased H^+^ flux regardless of whether the primary extracellular pH buffer was 26 mM bicarbonate or 1 mM HEPES, and persisted when atmospheric levels of CO_2_ were replaced by oxygen. Like retinal Müller cells, 100 μM adenosine did not alter extracellular H^+^ flux from baseline levels, and ATP-mediated increases in H^+^ flux were inhibited by the P2 antagonists suramin and PPADS, again suggesting activation of ATP receptors. Also like Müller cells, the increased level of H^+^ flux initiated by extracellular ATP appeared to depend critically on increases in intracellular calcium released from intracellular stores. Consistent with many earlier findings, extracellular ATP induced an intracellular rise in calcium in cultured astrocytes, and ATP-induced rises in both calcium and H^+^ efflux were significantly attenuated when calcium re-loading into internal stores was inhibited by thapsigargin. Figure 8C shows an ATP-elicited increase in H^+^ efflux from one astrocyte first in ordinary Ringer’s solution and then from the same cell after application of 1 μM thapsigargin. The response in thapsigargin was virtually abolished, while the response to a second application of 100 μM ATP in plain Ringer’s solution without thapsigargin present elicited an increase in H^+^ flux that was statistically indistinguishable from an initial application of ATP in control cells.

One interesting difference between ATP-elicited increases in H^+^ flux from astrocytes compared to Müller cells was the relative lack of dependence of increases in H^+^ flux from astrocytes on extracellular sodium. Replacement of extracellular sodium with choline did not significantly reduce the ATP-induced increases in H^+^ flux from cultured astrocytes, and increases in H^+^ flux were not significantly affected by the addition of EIPA, a blocker of Na^+^/H^+^ transport activity. One attractive alternative mechanism is the activity of monocarboxylate transporters, thought to play an important role in enabling astrocytes to provide lactate to neurons for their energy needs and which require co-transport of lactate with H^+^ (Pierre and Pellerin, 2005; Jha and Morrison, 2018, 2020). The release of lactate would likely be greatest when neuronal activity is highest, coinciding with the highest levels of extracellular ATP co-released with neurotransmitters when numerous vesicles fuse. Another potential mechanism involves calcium-stimulated fusion of vesicles with the plasmalemma of glia (see Malarkay and Parpura, 2011); H^+^ ions present in such vesicles could contribute to an extracellular acidification. V-ATPases appear not to be major contributors, as the addition of bafilomycin A_1_ failed to significantly alter ATP-induced increases in astrocytes. In 11 astrocytes tested, application of 100 μM ATP increased H^+^ flux to 183 ± 29 μV from an initial H^+^ standing flux of 87 ± 26 μV (*P* < 0.0001). The same cells were then exposed to 2 μM bafilomycin for several minutes, which has been reported to inhibit vacuolar ATPases with an IC_50_ of about 0.5 nM (Al-Fifi et al., 1998). 100 μM ATP again significantly increased the size of the H^+^ flux signal to 167 ± 26 μV from an initial standing flux in the presence of bafilomycin of 96 ± 26 μV (*P* = 0.0006). Neither the standing flux (*P* = 0.1854) nor the ATP response (*P* = 0.0768) was significantly different in bath solutions with and without bafilomycin A_1._ A previous study examining acid efflux from astrocytes cultured from the neopallium of 1 day old rats using microphysiometry, an entirely different technique (McConnell et al., 1992), also indicated that extracellular ATP induces acid efflux (Dixon et al., 2004). In that study, it was noted that cariporide, an inhibitor of Na^+^/H^+^ exchange, suppressed the initial portion of ATP-induced H^+^ efflux, and the authors argued for a role for Na^+^/H^+^ transport. However, the 5 μM cariporide used depressed ATP-elicited H^+^ efflux measured by microphysiometry by only about 30%, indicating that the majority of H^+^ release was not due to NHE1 exchange. Given the nature of microphysiometry experiments (measuring the sum of acid released by all cells present in a culture dish), it is possible that some of the measured H^+^ release might have come from cells other than astrocytes. One advantage of self-referencing H^+^ microelectrodes is the ability to restrict measurements to single cells possessing a clear astrocytic morphology. On the other hand, the high spatial resolution of H^+^-selective microelectrodes also means that H^+^-selective microelectrodes may have been consistently positioned at regions of the cell low in Na^+^/H^+^ activity and might have missed higher levels at other locations. Another difference in methodology is that experiments conducted with microphysiometry were done on confluent cell cultures grown for 11 days, while those employing self-referencing were done on cells examined at earlier times prior to confluence, and the physiological properties of the astrocytes might differ in the two sets of culturing conditions. Nevertheless, the main conclusion—that ATP elicits increases in H^+^ flux from cultured astrocytes—is supported by both the earlier microphysiometry studies as well as studies using self-referencing H^+^-selective electrodes.

## Discussion

### Implications of ATP-Elicited H^+^ Efflux From Glial Cells

We have seen above that H^+^ is one of the most potent modulators of synaptic transmission occurring naturally in the nervous system, acting as an inhibitor of voltage-gated calcium channels, an inhibitor of such post-synaptic receptor proteins as NMDA glutamate receptors, and facilitating removal of neurotransmitters from the synaptic cleft. We have also seen that extracellular ATP induces H^+^ release from retinal radial glial cells as well as astrocytes cultured from several areas of the brain. In this section, we examine several hypotheses for the role of alterations in extracellular H^+^ in regulating synaptic transmission within the vertebrate retina and then broaden our scope to discuss implications of ATP-elicited H^+^ efflux from glial cells for such phenomena as epilepsy, migraines, and electrical patterns of brain rhythms.

The high sensitivity of photoreceptor calcium channels to changes in extracellular H^+^ has led to the hypothesis that the surround portion of the classic center-surround receptive field of retinal neurons may result from alterations in extracellular acidity—but mediated by retinal horizontal cells (Thoreson and Mangel, 2012; Kramer and Davenport, 2015). In one common version of this hypothesis, glutamate released by photoreceptors depolarizes horizontal cells, leading to increased release of H^+^ from horizontal cells and inhibition of photoreceptor calcium channels with the consequent reduction in glutamate release. Strong evidence supporting this hypothesis comes from experiments employing CalipHlourin, a novel sensor developed to detect changes in extracellular H^+^ at synaptic terminals of vertebrate photoreceptors (Wang et al., 2014). CalipHluorin is a pH-sensitive form of GFP (pHluorin) attached to the extracellular N terminus of the α2δ4 subunit of L-type calcium channels that is selectively expressed in cone photoreceptors, allowing measurements of extracellular H^+^ at the level of photoreceptor synaptic terminals. Extracellular levels of H^+^ were highest with retinal slices in the dark, and light stimuli induced an extracellular alkalinization. The light-induced effects on extracellular H^+^ became larger as light stimuli were varied from small to a larger diameter, a characteristic expected from the large receptive field size of horizontal cells. In addition, activation by FMRF-amide of genetically modified zebrafish horizontal cells containing FMRF-amide receptors induced an extracellular acidification reported by alterations in CalipHlourin fluorescence.

However, experiments measuring changes in extracellular H^+^ flux from horizontal cells with self-referencing H^+^-selective electrodes consistently reveal that depolarization of isolated horizontal cells by glutamate or high extracellular potassium induces an extracellular alkalinization, not the expected acidification called for by the original hypothesis (Molina et al., 2004; Kreitzer et al., 2007, 2012; Jacoby et al., 2012). Data suggest that the extracellular alkalinization stems from activation of plasma membrane calcium pumps (PMCA), which take in two extracellular H^+^ for each calcium extruded. In this model, depolarization of horizontal cells promotes the influx of calcium through voltage-gated calcium channels and calcium-permeable AMPA glutamate receptors, with consequent activation of PMCA pumps to extrude accumulated intracellular calcium, resulting in an extracellular alkalinization. Experiments examining changes in fluorescence of the H^+^ reporter HAF when restricted to the plasma membrane of horizontal cells confirmed that activation of horizontal cells by potassium and glutamate induced an extracellular alkalinization, and further demonstrated that this alkalinization occurred over the entire external cell surface, with no isolated hot spots of acidification (Jacoby et al., 2014). It is also worth noting that the maximal size of the ATP-elicited extracellular acidification measured from isolated Müller cells is almost an order of magnitude larger than the biggest extracellular alkalinization detected from activated horizontal cells using identical methods.

**Figure 9:**
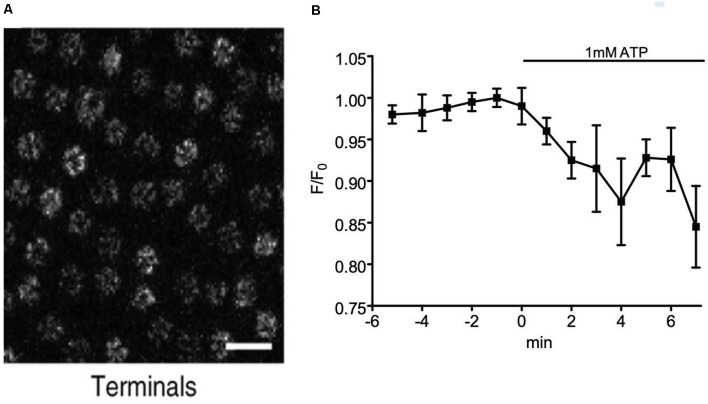
Changes in extracellular H^+^ at the level of the synaptic terminals in the retinal slice of a zebrafish. **(A)** Fluorescent micrograph showing the pattern of expression of the extracellular pH indicator CalipHluorin at the level of the synaptic terminals of cone photoreceptors of the zebrafish retina. To produce CalipHluorin, the H^+^-sensitive GFP pHlourin is fused to the extracellular N terminus of the α2δ4 subunit of the L-type calcium channel. From Wang et al. (2014). **(B)** Addition of 1 mM extracellular ATP induced a decrease in fluorescence reported by CalipHluorin indicative of extracellular acidification at the level of the outer synaptic (plexiform) layer from five zebrafish retinal slices. Measurements were made as described in detail in Wang et al. (2014). From data presented in abstract form by Malchow et al. (2018).

At the time the original experiments with CalipHluorin were done (Wang et al., 2014), it was not known that extracellular ATP could induce alterations in extracellular H^+^ via activation of Müller cells. Another explanation for the observed alterations in extracellular H^+^ previously reported by CalipHluorin, then, is that activation of retinal neurons leads to release of neurotransmitter as well as ATP, leading to ATP-elicited activation of Müller cells and consequent release of H^+^ from the radial glial cells of the retina. Synaptic vesicles possess high concentrations of ATP which can be released to the extracellular space by fusion of vesicles with the plasma membrane (for review, see Pankratov et al., 2006; Abbracchio et al., 2009; Burnstock and Verkhratsky, 2012), and the higher levels of H^+^ observed in the dark may reflect H^+^ extrusion by Müller cells in response to ATP released upon fusion of synaptic vesicles with the plasma membrane. Activation of genetically modified horizontal cells with FMRF-amide would likely result in a strong depolarization and greatly increased intracellular calcium levels inside these cells with consequent stimulation of fusion of synaptic vesicles and release of vesicular ATP, which again would result in increased stimulation of ATP-induced release of H^+^ from Müller cells. The increase in the size of the extracellular acidification with increasing size of light stimulus, while consistent with the relatively large receptive field of horizontal cells, might reflect activation of glial cells adjacent to horizontal cells, effectively mimicking the receptive field size of horizontal cells. Additional evidence in support of the contention that the extracellular changes in H^+^ reported by CalipHluorin might come from radial glial cells is illustrated in Figure 9. The left-hand portion of the figure shows staining of CalipHluorin seen at the level of synaptic terminals of photoreceptors (Figure 9A), while the right shows data that application of 1 mM extracellular ATP on to zebrafish retinal slices induces extracellular acidification at the synaptic terminal as reported by CalipHlorin (Figure 9B; presented in abstract form in Malchow et al., 2018). The extracellular acidification detected by CalipHluorin is consistent with increases in extracellular levels of H^+^ induced by ATP as detected using self-referencing H^+^-selective electrodes at the level of the outer plexiform (synaptic) layer of retinal slices of the tiger salamander as shown in Figure 4.

We envision, then, that “feedback” is being provided continually at photoreceptor synaptic terminals by the release of H^+^ from radial glial cell processes activated by extracellular ATP likely co-released with glutamate during fusion of synaptic vesicles. Thus, many of the findings examining feedback onto vertebrate photoreceptors initially ascribed to neurons may reflect at least in part glial-mediated inhibition of synaptic transmission *via* release of H^+^. To be sure, the evidence that H^+^ plays a role in regulating synaptic transmission at the OPL is strong. DeVries (2001) and Hosoi et al. (2005) have presented compelling data that H^+^ co-released from photoreceptor synaptic vesicles acts as a highly transient (several msec) but quite potent inhibitor of neurotransmitter release from photoreceptors. On a greater scale of time and space, Hirasawa and Kaneko (2003) and Cadetti and Thoreson (2006) demonstrated alterations in the voltage-dependent calcium conductance of cone photoreceptors consistent with effects mediated by extracellular H^+^. Initially attributed to horizontal cells, we propose an effect secondarily mediated by activation of Müller cells *via* extracellular ATP with consequent extrusion of H^+^, in part through activation of Na^+^/H^+^ exchange activity.

Measurements of extracellular H^+^ flux in retinal slices indicate that extracellular ATP induces extracellular acidification not only at the outer synaptic layer of the retina but also at the more complex inner plexiform (synaptic) layer, where bipolar cells make synaptic connections with amacrine and ganglion cells. This suggests that inhibition of synaptic transmission by ATP-elicited changes in extracellular H^+^ may extend to this area of extensive signal processing as well. Modulation of neuronal responses in the inner retina by activation of glial cells has been reported previously (Newman and Zahs, 1998; Newman, 2003). At least part of the modulation resulting from activation of Müller cells was ascribed to the actions of adenosine converted from extracellular ATP by extracellular ATPases acting on receptors present on the neurons (Newman, 2003). In this latter study, extracellular recording solutions contained 10 mM HEPES, a concentration expected to significantly blunt changes in extracellular H^+^ that might result from stimulation of Müller cells and reduce the impact of H^+^-dependent inhibition of synaptic transmission. We hypothesize that under conditions in which normal concentrations of the physiological buffer bicarbonate are used, alterations in H^+^ resulting from release *via* stimulation of Müller cells might further contribute to the modulation of neuronal signals within the inner synaptic layer of the retina.

Circadian rhythms in levels of extracellular H^+^ in the retina have been detected as well as circadian changes in the gain of synaptic transfer at the outer synaptic layer of the retina. Measurements of extracellular H^+^ in isolated retinae of goldfish and rabbits using H^+^-selective microelectrodes revealed a significantly higher level of H^+^ in the subjective night compared to subjective daytime (Dmitriev and Mangel, 2000, 2001; Mangel, 2001). Additional experiments examining light-induced responses of goldfish horizontal cells demonstrated that during the subjective day, the cone photoreceptor-to-cone-driven horizontal cell transfer function exhibited an asymptotic synaptic gain of about 1.5, while the gain at subjective night decreased to about 0.3 (Ribelayga and Mangel, 2019). In the dark of subjective night, photoreceptors would be expected to be in their most depolarized state, and theoretically constantly liberating high amounts of glutamate. It has always been a mystery as to why the release of neurotransmitters from photoreceptors should be highest in the dark, when we are asleep, and why this expected continuous release of neurotransmitters would not induce glutamate-mediated neuronal excitotoxicity, as observed in other areas of the brain when high levels of glutamate are present. We propose that glutamate release from tonically depolarized photoreceptors and other neurons is accompanied by co-release of ATP, leading to extracellular acidification mediated by activation of Müller cells. We further hypothesize that this release of H^+^ from Müller cells acts as a form of automatic gain control, decreasing calcium influx into synaptic terminals and limiting neurotransmitter release from retinal neurons, and decreasing synaptic gain without significantly altering the ability of light to alter the voltage of photoreceptive cells themselves.

A circadian rhythm in levels of extracellular adenosine has been reported in the retina as well, with the highest levels occurring at night under dark adapted conditions (Ribelayga and Mangel, 2005; Cao et al., 2020). This distribution would be consistent with high levels of extracellular ATP at the same time—at subjective night and in the dark—since the enzymatic breakdown of ATP by extracellular ectonucleases would result in the production of extracellular adenosine. Circadian patterns in levels of adenosine have also been detected in other areas of the brain (Huston et al., 1996; Murillo-Rodriguez et al., 2004). Both adenosine and astrocytes have long been implicated with the regulation of sleep (see Bjorness and Greene, 2009; Blutstein and Haydon, 2013; Bjorness et al., 2016; Greene et al., 2017; Haydon, 2017). There is also evidence for a circadian rhythm associated with ATP in the suprachiasmatic nucleus as well as with cultured astrocytes; moreover, levels of extracellular ATP are associated with increases in intracellular calcium in astrocytes (Womac et al., 2009; Burkeen et al., 2011; Marpegan et al., 2011). Based on our data demonstrating ATP-induced H^+^ efflux from cultured astrocytes from rat cortex and mouse hippocampus and their dependence upon intracellular calcium, it seems reasonable to hypothesize that alterations in extracellular acidity mediated by glial cells could play an important role in inducing sleep. Sleep and wakefulness are highly complex processes involving large networks of neurons and also appear to involve a large number of transmitter chemicals and modulators (see Luppi and Fort, 2011; Holst and Landolt, 2018; Landolt and Dijk, 2019). Given that glial cells are widely distributed and embedded in all of these networks with their fine processes wrapping and enveloping virtually every synapse, and further given the exceptional sensitivity of synaptic transmission to changes in extracellular H^+^, it may be that glial release of H^+^ plays a role in regulating waking and sleeping and driving the transitions between these behavioral states.

The various stages of sleep are well known to be accompanied by alterations in brain rhythms measured with extracellular electrodes (EEG). Increases in the efflux of extracellular H^+^ mediated by alterations in intracellular calcium within glial cells might provide the physical link by which glial cells could induce changes in both brain states as well as corresponding electrical activity measured with an EEG. The altered threshold for initiation of neurotransmitter release anticipated when levels of extracellular H^+^ are varied, as well as alterations in postsynaptic receptor activation and the speed with which released neurotransmitters are taken up, could influence the rhythmic pattern of firing of ensembles of neurons, leading to changes noted in EEG recordings. Alterations in brain rhythms measured by an EEG are also well known to occur upon administration of anesthetics; indeed, the dose, strength, and impact of an anesthetic are often assessed by the variation in EEG signals that are recorded. While the cellular and molecular mechanism of action of some anesthetics are well known, there remain a number of questions about the mode of action of some important and widely used compounds such as general anesthetics, for which the mechanism is not yet entirely clear. While most studies have naturally focused on the direct effects of anesthetics on neurons, it is plausible that some of the effects of some anesthetics might result from effects at the level of the glial cells. Given the high sensitivity of synaptic transmission to alterations in extracellular H^+^, anesthetic actions resulting in the release of H^+^ from glia could account for some of the sedative effects of such compounds.

**Figure 10:**
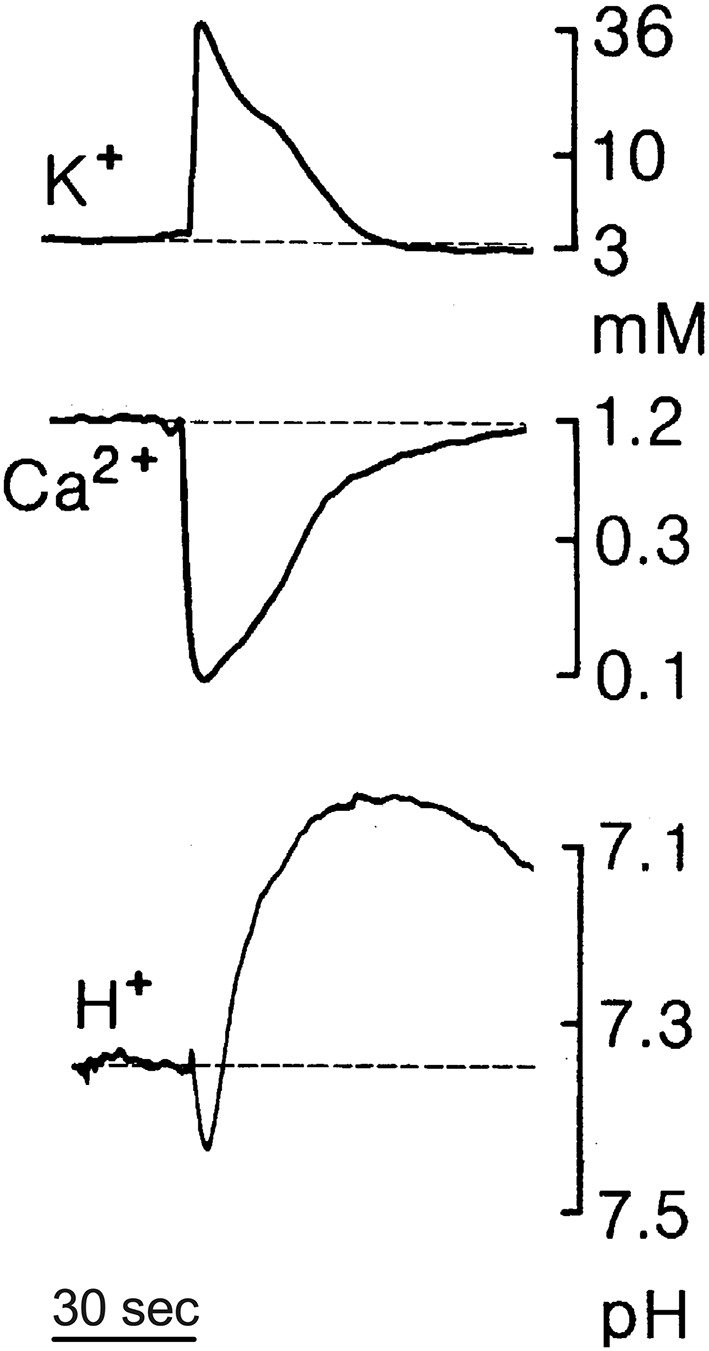
Changes in extracellular levels of potassium (K^+^), calcium (Ca^2+^), and H^+^ during spreading depression in the rat cerebellum measured using ion-selective microelectrodes; based on data by Charles Nicholson presented in Martins-Ferreira et al. (2000). Spreading depression was initiated by brief trains of local surface stimuli. Notice the biphasic change in extracellular levels of H^+^, with an initial small transient alkalinization followed by a larger and more prolonged extracellular acidification.

We also hypothesize that release of H^+^ from glia elicited by extracellular ATP provides the physical link underpinning the profound inhibition of electrical activity encountered in the phenomenon commonly referred to as spreading depression. Characterized in part by a slowly expanding wave of profound depression of spontaneous and evoked electrical activity in the nervous system that can last minutes at any one point, the phenomenon of spreading depression has been detected in the cortex, hippocampus, cerebellum, and other areas and has also received extensive study in the retina, where it is marked by readily observed optical changes (for review, see Martins-Ferreira et al., 2000; Gorji, 2001; Cozzolino et al., 2018). Large changes in the extracellular concentrations of a number of different ions including H^+^ occur during spreading depression (Mutch and Hansen, 1984; Nicholson, 1984; Rice and Nicholson, 1988; Martins-Ferreira et al., 2000; Menna et al., 2000; Tong and Chesler, 2000). Figure 10 shows an example of changes in extracellular levels of potassium, calcium, and H^+^ detected during spreading depression in the rat cerebellum. Unlike the monophasic changes in extracellular potassium and calcium, alterations in extracellular H^+^ during spreading depression are biphasic, with an initial small transient alkalinization followed by a larger and more long-lasting extracellular acidification. The slow wave of profound inhibition during spreading depression is preceded by a slow “spreading depolarization” accompanied by a burst of neuronal electrical activity (Charles and Baca, 2013; Cozzolino et al., 2018). This initial high level of neuronal activation should induce large increases in calcium entering neurons through activation of voltage-gated calcium channels in presynaptic neuronal terminals as well as through activation of receptors that are calcium-permeant such as NMDA receptors in postsynaptic processes. Indeed, another hallmark of ionic changes occurring during spreading depression is depletion of extracellular calcium, as illustrated in Figure 10, consistent with calcium entering neurons during the initial depolarization event (Kraig and Nicholson, 1978; Nicholson, 1984; Martins-Ferreira and do Carmo, 1987; Martins-Ferreira et al., 2000). The contribution of Na^+^/Ca^2+^ exchange to the restoration of normal levels of intracellular calcium in neurons is likely to be compromised since extracellular sodium also plummets during spreading depression. The reestablishment of pre-stimulus levels of intracellular calcium in neurons is thus likely to depend critically upon the activity of the plasmalemma calcium pump, which takes in two extracellular H^+^ for each calcium extruded (Thomas, 2009). We envision that the initial extracellular alkalinization detected in spreading depression results from increased activity of PMCA pumps on neurons accumulating extracellular protons while extruding calcium ions following the burst of neuronal activity at the beginning of the event. Increases in extracellular ATP by as much as 100 μM have also been reported to occur during spreading depression (Schock et al., 2007), which based on our studies should activate pronounced H^+^ extrusion from glial cells into the extracellular space, likely resulting in the secondary prolonged acidification observed during spreading depression. We further hypothesize that glial-mediated extrusion of H^+^ is essential in restoring calcium levels in neurons to pre-stimulus conditions. Thus, rather than reflecting a nebulous and hazy increase in extracellular H^+^ attributed in the past to general increases in overall cellular metabolism, we propose that the secondary increase in extracellular H^+^ is mediated specifically by glial extrusion of H^+^ initiated by high levels of extracellular ATP, and is likely essential for providing extracellular H^+^ needed for restoration of normal calcium levels in neurons *via* the action of PMCA pumps. This same glial-mediated increase in H^+^ would also depress additional neurotransmission by blocking voltage-gated calcium channels and certain post-synaptic responses, and enhance the uptake of neurotransmitters from the extracellular space, leading to the profound inhibition of spontaneous and evoked responses observed during spreading depression.

Spreading depression has long been associated with slow waves of increased intracellular calcium in glial cells (Basarsky et al., 1998; Kunkler and Kraig, 1998; Peters et al., 2003; Chuquet et al., 2007; Charles and Brennan, 2009), consistent with the calcium-dependence observed for increases in glial cell-mediated H^+^ extrusion. Glial calcium waves have also been tied to increases in extracellular ATP, and it has been proposed that glial-mediated release of extracellular ATP may play a role in the generation and propagation of such spreading waves (Newman and Zahs, 1997; Guthrie et al., 1999; Newman, 2001; Weissman et al., 2004; Hoogland et al., 2009). Slow propagating waves of cortical activity have also been associated with migraines, and a link between the phenomenon of spreading depression and the occurrence of certain migraines has long been suspected (see Gorji, 2001; Rogawski, 2008; Eikermann-Haerter and Ayata, 2010; Charles and Baca, 2013; Eikermann-Haerter et al., 2013; Cozzolino et al., 2018; Harriott et al., 2019; Takizawa et al., 2020). Migraines are a heterogenous set of headache-related maladies reported to occur in many different parts of the brain and appear to involve widespread changes in multiple neuronal pathways and involve a number of neurochemical mediators [Davidoff, 2002; Charles, 2013; Headache Classification Committee of the International Headache Society (IHS) (2013)]. The aura perceived in certain migraines has been suggested to result from the initial spreading depolarizing wave that precedes the profound suppression of neuronal responses characteristic of spreading depression (Lauritzen, 1994; Cui et al., 2014; Charles, 2018; Major et al., 2020; Harriott et al., 2021). The pain in certain migraines following an aura might then result in part from the excessive and prolonged release of H^+^ from glia during the period of synaptic inhibition occurring during the depression that follows the spreading depolarization. H^+^ has been strongly implicated in pain pathways, and an excess of released H^+^ from glial cells could potentially act on ASICs (acid sensing ion channels) and TRP (transient receptor potential) receptors to induce pain; indeed, both ASICs and TRP channels have specifically been proposed as targets for pharmacological approaches to reduce pain associated with migraine headaches (see Holland et al., 2012; Dussor, 2015, 2019; Benemei and Dussor, 2019; Takizawa et al., 2020).

A potential association between migraine and epilepsy has also long been suspected (see Post and Silberstein, 1994; De Simone et al., 2007; Parisi et al., 2008; Rogawski, 2008; Kasteleijn-Nolst Trenité et al., 2010; Keezer et al., 2015; Zarcone and Corbetta, 2017; Çilliler et al., 2017). Indeed, as pointed out by a number of investigators, the original description of spreading depression by Leão (1944) was done in the context of studies designed to induce experimental epilepsy (see Somjen, 2005; Teive et al., 2005; Cozzolino et al., 2018). We propose that the release of H^+^ by glial cells constitutes a key and essential protective mechanism by which the nervous system normally prevents the development and spread of excessive neuronal stimulation that is a hallmark of epileptiform activity. In this view, the application of supra-normal stimulation such as that employed by Leão induces an initial massive depolarization of large numbers of neurons that in turn leads to high levels of extracellular ATP and consequent increases in intracellular calcium in neighboring glial cells. The high calcium in glial cells promotes the release of H^+^ into the extracellular milieu, inhibiting further synaptic transmission by H^+^-induced inhibition of voltage-gated calcium channels and certain post-synaptic receptors, as well as accelerating the removal of neurotransmitters *via* H^+^-dependent uptake and also facilitating removal of intracellular calcium from over-activated neurons. Thus, we envision a negative feedback loop—a reset switch, if one will—whereby ATP-elicited H^+^ extrusion by glial cells shuts down and prevents excess neuronal activity for a time while promoting a restoration of pre-stimulus levels of extracellular and intracellular ions and neurotransmitters. We hypothesize that the initial high levels of extracellular ATP stimulating H^+^ release from glial cells likely come from ATP co-packaged with neurotransmitters in synaptic vesicles and that this is then augmented by ATP released by glial cells themselves, resulting in the wave of spreading depression mediated by extracellular H^+^. The wave of spreading depolarization elicited by supra-normal stimuli, or that might occur in certain epilepsies upon excessive neuronal activity in potentially sensitive foci, would be expected to elicit a following wave of profound neuronal depression mediated by H^+^ release from glial cells.

Epilepsies, like migraines, are a diverse set of nervous system abnormalities likely to have a number of different etiologies (see Chang and Lowenstein, 2003; Stafstrom and Carmant, 2015). With that important caveat in mind, it is notable that a sizable literature links astrocytes with epileptiform activity (see de Lanerolle et al., 2010; Carmignoto and Haydon, 2012; Vargas-Sánchez et al., 2018; Patel et al., 2019; Binder and Steinhäuser, 2021), and with purinergic signaling (Kumaria et al., 2008; Engel et al., 2016; Nikolic et al., 2020) as well as Na^+^/H^+^ transport (Gu et al., 2001; Zhao et al., 2016; Fliegel, 2020). We propose that excessive levels of neuronal activity associated with certain epilepsies might occur in conditions in which the signal transduction pathway leading to H^+^ release from glial cells has been compromised, or when glial processes are aberrantly too far away from sites of synaptic activity for H^+^ released by glia to inhibit neuronal activity. Tuberous sclerosis complex is one such epileptic condition in which this may be the case. This is a genetic disorder associated with the mTOR signaling pathway that results in epileptic activity in most individuals afflicted with the disease and is characterized by significant alterations in the relationships between glia and neurons (reviewed in Curatolo et al., 2002, 2015; Chu-Shore et al., 2010; Sofroniew and Vinters, 2010; Zimmer et al., 2020). The morphological and functional properties of the glial cells themselves are significantly distorted in this disease; for example, giant glial cells with exceptionally large cell bodies possessing relatively sparse fine processes are common (see Mizuguchi and Takashima, 2001; Boer et al., 2008; Grajkowska et al., 2010; Zimmer et al., 2020). Tuberous sclerosis complex has also been associated with disruptions in the balance of synaptic excitation and inhibition (Dooves et al., 2021). We hypothesize that the aberrant positioning and properties of glial cell fine processes in this condition leads to an inability of these cells to provide essential H^+^-mediated inhibition of synaptic activity needed to prevent epileptiform activity, as well as inhibiting adequate removal by H^+^-dependent transporters of neurotransmitters released into the synaptic cleft. We also predict that epileptic foci in other epilepsies might result from similar abnormalities in H^+^-mediated regulation of synaptic activity and neurotransmitter uptake by glial cells. Excessive excitation at such foci could result either from compromises in the signal transduction pathway leading to H^+^ release or alterations in the spatial relationship between glial cells and neurons that prevent H^+^ released from glial cells from reaching synaptic terminals. A previous study observed close correlations of neuronal bursting with changes in intracellular calcium in glial cells and concluded that increases in calcium in glial cells were the cause of epileptic-like behavior resulting from glial-mediated release of glutamate (Tian et al., 2005). However, others have presented data demonstrating that astrocytic glutamate is not necessary for the generation of epileptiform activity in hippocampal slices and have argued that the discharge of action potentials in neurons, rather than astrocytic release of glutamate, is the key driver of epileptiform activity (Fellin et al., 2006). More recent studies have suggested that glial calcium waves are triggered by seizure activity and are not essential for epileptogenesis (Baird-Daniel et al., 2017). Our model would predict close correlations of glial cell increases in intracellular calcium with increases in neuronal activity, but with the calcium-dependent release of H^+^ from glia acting as a response to reduce neuronal activity to suppress excessive stimulation.

It is interesting to note that CA inhibitors have long been employed in the treatment of epilepsy (see Bergstom et al., 1952; Ansell and Clarke, 1956; Reiss and Oles, 1996; Thiry et al., 2007; Mishra et al., 2021). Indeed, intravenous administration of the CA inhibitor acetazolamide induced a significant extracellular acidosis in the brains of anesthetized cats (Heuser et al., 1975). Extracellular CAs are known to be present in the brain interstitial space and have been suggested to play important roles in regulating extracellular H^+^ levels (Chen and Chesler, 1992, 2015; Tong et al., 2000; Makani and Chesler, 2010). We envision that CA inhibitors crossing the blood-brain barrier, entering the brain, and reaching synapses in sufficient concentrations might exert their salutary actions primarily by inhibiting extracellular CA activity. Drugs that inhibit the activity of extracellular CAs near synaptic terminals could significantly impact the magnitude and time course of synaptic inhibition mediated by H^+^ released from glial cells. If a little is beneficial, however, more may not be better, since, at higher concentrations, acetazolamide and comparable CA inhibitors would likely cross the plasma membrane of glial cells and compromise the activity of intracellular CAs, reducing the amount of intracellular H^+^ produced and secreted. This expected inverted U-shaped dose-response relationship, along with side effects resulting from these drugs on CAs present throughout the body, may be why such drugs are not the first choice for the treatment of epilepsy.

In response to injury and disease, glial cells undergo reactive gliosis, a complex and broad term that includes a host of morphological, molecular, and functional alterations (Escartin et al., 2021). The phenomenon has been described as a finely graded continuum of alterations that range from reversible changes in gene expression and cell hypertrophy to scar formation with permanent alterations in overall morphological arrangements (see Anderson et al., 2014; Burda and Sofroniew, 2014; Pekny et al., 2014; Robel and Sontheimer, 2016; Liddelow and Barres, 2017). In conditions where glial cells form scar-like structures, such as occurs in transection of the spinal cord, it has proven difficult to encourage neurons to grow through the scar. A number of studies have suggested that reactive glia have increases in the frequency and amplitude of alterations in intracellular calcium (for review, see Agulhon et al., 2012; Shigetomi et al., 2019; Verkhratsky, 2019). If such increased levels of glial intracellular calcium also result in increased extrusion of H^+^, consequent elevations in overall extracellular H^+^ could be one factor limiting the growth and extension of neurons through glial scars. The influx of calcium into specific portions of neurites is thought to play a key role in guiding the growth and extension of neurites in specific directions (Kater and Mills, 1991; Henley and Poo, 2004; Rosenberg and Spitzer, 2011; Gasperini et al., 2017). High levels of extracellular H^+^ at the site of a glial scar would then be expected to prevent calcium channels in nearby neurites from opening, thus impeding neurite outgrowth. Thus, enhanced extrusion of H^+^ by reactive glial cells could be one mechanism hindering the reestablishment of neuronal connections in injured or diseased states, and treatments designed to reduce extracellular levels of H^+^ could potentially improve the reestablishment of neuronal growth and connectivity.

## Towards the Future

To sum up, small alterations in H^+^ are exceptionally potent inhibitors of synaptic transmission, inhibiting voltage-gated calcium channels in presynaptic cells and certain neurotransmitter receptors on postsynaptic cells, and playing a critical role in the uptake of neurotransmitters as well as facilitating extrusion of intracellular calcium *via* PMCA pumps. Quite low concentrations of extracellular ATP promote H^+^ extrusion from retinal radial glial cells. The ATP-elicited H^+^ efflux is observed in Müller cells isolated from organisms as evolutionarily distant as lamprey, skate, tiger salamander, rat, monkey, and human, implying an important role conserved throughout vertebrate evolution. Extracellular ATP also elicits H^+^ efflux from astrocytes cultured from mouse hippocampus and rat cortex, suggesting that ATP-elicited H^+^ efflux is likely a general property of radial glia and astroglial cells. The fine processes of glial cells wrap and extensively envelop nervous system synapses, and thus release of H^+^ by glia upon activation by extracellular ATP is well poised to regulate synaptic activity. This leads us to postulate an important role for glial cell-released H^+^ in modulating neuronal activity in both normal and aberrant conditions. We envision that ATP-elicited H^+^ flux from glia provides essential inhibition throughout the nervous system, and this inhibition prevents overexcitation of synaptic circuits. We also postulate that the release of H^+^ by glia is the molecular mechanism underpinning the phenomenon of spreading depression and is a key and essential factor in preventing epileptiform activity. If this is correct, the broad extent of spreading depression and its observation in many areas of the nervous system would be consistent with our contention that ATP-elicited H^+^ release is a general property of many glia. The similarity in the characteristics of spreading depression and migraine noted by others also leads us to suggest a potentially causative role for excessive glial cell-mediated H^+^ release in the etiology of migraine. We further propose that disruptions in either the signal transduction pathway leading to H^+^ efflux from glia or the positioning of glial processes away from the synapse, may prevent adequate inhibition of synaptic activity by H^+^ efflux from glia and lead to the development of epileptiform activity.

We hypothesize that under normal levels of neuronal activity, synaptic activity is adjusted by the glial cell-mediated release of H^+^ on a synapse-by-synapse basis. This would imply that the fine processes of individual glial cells act independently, with local variations of intracellular calcium leading to localized alterations in H^+^ flux. Studies examining the three- dimensional pattern of calcium dynamics in awake animals and brain slices have demonstrated that calcium increases in astrocytes are scattered throughout the cell, are highly compartmentalized within predominantly local regions, and are heterogeneously distributed regionally and locally (Bindocci et al., 2017; Savtchouk et al., 2018). Additionally, these studies indicate that astrocytes can respond locally to quite minimal axonal firing with time-correlated changes in intracellular calcium. This suggests that potential regulation of neurotransmitter release by H^+^ extruded from glia could be highly localized and that inhibition provided by H^+^ release initiated by local increases in calcium may modulate synaptic strength at a synapse-by-synapse scale. The thousands of fine processes from astrocytes wrapping various separate synapses could thus potentially act independently from one another, with ATP released at just that synapse acting to locally influence synaptic strength by the highly localized release of H^+^.

When excitation is intense, we hypothesize that waves of calcium throughout glial cells are generated that induce increases in extracellular H^+^ at many synapses simultaneously, resulting in the coordinated shutdown of neuronal activity so characteristic of spreading depression. Such waves of calcium may also induce the release of ATP by glial cells themselves, leading to further lateral spread of ATP-elicited H^+^ inhibition. An important question to address in future experiments will be the mechanism by which local H^+^-mediated inhibition provided by calcium changes in the fine processes of glial cells, transitions to waves of calcium within and across glial cells, leading to inhibition over larger spatial and temporal scales. One possibility is that excess neuronal stimulation in a local area leads to large increases in extracellular potassium and perhaps glutamate, and a concomitantly significant decrease in extracellular calcium as it enters neurons through voltage-gated calcium channels on presynaptic terminals and calcium-permeant receptors on post-synaptic neurons. The high level of extracellular potassium and glutamate resulting from the excessive neuronal activity then might lead to depolarization of neighboring neurons, consistent with the wave of spreading depolarization that precedes the profound depression of responses in the later stage of spreading depression. Low levels of extracellular calcium induce, almost paradoxically, significant increases in levels of intracellular calcium in astrocytes, as well as enhancing the intracellular calcium changes elicited by extracellular ATP (Zanotti and Charles, 1997). The increases in intracellular calcium in astrocytes induced by decreases in extracellular calcium may be mediated by store-operated Ca^2+^ release-activated Ca^2+^ (CRAC) channels (Toth et al., 2019). The lowered levels of extracellular calcium induced by excessive neuronal activation might then act as a signal that leads to increases in intracellular calcium in glial cells and an increase in calcium-dependent H^+^ efflux from glial cells.

The overall impact of H^+^ released by glial cells on transmission at synaptic terminals will depend on a complex number of factors, including the amount and duration of release of H^+^, the distance of H^+^ extrusion sites on glia from synaptic terminals, the concentration, strength, and nature of buffers for H^+^ present, the location and amount of bicarbonate transporters, and the location and amount of CA present. Our model for activation of H^+^ efflux by extracellular ATP in retinal Müller cells calls in part for the production of both H^+^ and HCO_3_ *via* enzymatic conversion of CO_2_ mediated by the high concentrations of intracellular CA present in glia. Studies have shown that the bulk of HCO_3_ transport activity occurs at the basal end foot of Müller cells, significantly removed from the sites of most synaptic terminals (Newman, 1991; Kreitzer et al., 2017). Thus, bicarbonate that is produced within Müller cells would be largely secreted into the vitreous humor of the eye. On the other hand, ATP-elicited alterations in extracellular H^+^ measured using self-referencing electrodes show prominent extracellular acidifications at both the outer and inner synaptic layers of the retina (Tchernookova et al., 2018). We envision that activation of Müller cells by extracellular ATP leads to the production of both H^+^ and HCO_3_ and that a significant portion of the H^+^ generated is extruded by Na^+^/H^+^ exchange near synaptic terminals. The sodium internalized in this process, along with increased concentrations of HCO_3_ generated within the cells, would facilitate extrusion of HCO_3_ at the basal end-foot of the cells by Na-HCO_3_ cotransporters present in the end-foot; other types of HCO_3_ transporters might also facilitate extrusion of HCO_3_. We hypothesize, then, that generation of H^+^ and HCO_3_ occur together, but are exported largely at different locations in Müller cells, helping to preserve the overall level of intracellular pH inside the cell. It will be important in future studies to determine the relative spatial relationships between H^+^ extrusion and HCO_3_ transport within astroglial cells, as the overall effect of H^+^ extrusion on alteration of synaptic activity will likely be significantly different if HCO_3_ is secreted at the same location. The presence or absence of extracellular CA, and its precise localization, will also likely have a significant impact on the effects of H^+^ extruded by the glia. Even if H^+^ and HCO_3_ are extruded at the same site, the slow kinetics of the conversion of H^+^ and HCO_3_ to CO_2_ and water could result in a significant transient effect of H^+^ on synaptic transmission if extracellular CAs are low in number around the release site.

In future experiments to examine the effects of glial-released H^+^ on synaptic transmission, it will be critical to measure alterations in extracellular H^+^ upon selective stimulation of glia at the precise location where H^+^ is expected to exert its effects on synaptic transmission. The development of molecular sensors such as CalipHluorin—calcium channels engineered to express extracellular pHluorins as extracellular H^+^ sensors—now offers the opportunity to make such measurements (Wang et al., 2014). One can imagine the development of similar H^+^-sensitive tags expressed selectively in various cells and attached to other transporters or postsynaptic receptors to report alterations in extracellular H^+^ at these locations. A remaining challenge will be to selectively stimulate just the glia in various experimental preparations since many neurons possess receptors for extracellular ATP, and simple addition of ATP is thus likely to have multiple effects resulting from activation of many different sets of cells and cellular signaling transduction pathways. The use of DREADDS (designer receptors exclusively activated by designer drugs) offers one possible approach to solving this challenge, and these have successfully been incorporated into glial cells (see Xie et al., 2015; Bang et al., 2016; Losi et al., 2017; Yu et al., 2020). However, the use of DREADDS and the interpretations of data obtained from such experiments come with their own set of important caveats and concerns. One major concern is how closely the activation of DREADDS mimic the response initiated by naturally occurring receptors activated by normal physiological activators, in this case, the initiation of H^+^ flux by extracellular ATP. Our hypothesis that H^+^ release from fine branches and extensions of glial cells modulates synaptic activity at the level of individual synapses suggests a crucial role for microdomains in glial-mediated regulation of synaptic transmission by H^+^. It will be essential to ensure that the pattern of activation of, say, intracellular calcium by DREADDS expressed in glial cells mimics the natural alteration in calcium by ATP as closely as possible in both time and space.

Modulation of neuronal excitability by glial-induced changes in extracellular H^+^ has been hypothesized by others previously (see Ransom, 1992, 2000). The calcium-dependent increase in extracellular H^+^ flux initiated by extracellular ATP provides a straightforward molecular mechanism by which such modulation can take place. Our hypothesis calls for a broad, powerful, and essential regulation of synaptic transmission by H^+^ released by glia throughout the nervous system, with too little inhibition by glial H^+^ efflux leading potentially to epileptiform behavior, and too much leading to the misery of migraine. Our recent work demonstrating ATP-elicited H^+^ efflux from radial glia and astrocytes provides a specific molecular mechanism by which glia might provide feedback inhibition to limit synaptic transmission, and also provides a specific potential molecular link to spreading depression, migraine, and epilepsy. If this hypothesis is correct, it will be remarkable that an ion (H^+^), which generally receives little consideration from neurobiologists, released from a cell type that historically has been underappreciated in the area of neuroscience (glial cells), might have such an important and impactful role in the functioning of the nervous system that has been overlooked for so long.

## Data Availability Statement

The raw data supporting the conclusions of this article will be made available by the authors, without undue reservation.

## Ethics Statement

All animals were treated in accordance with the protocols approved by the Institutional Animal Care and Use Committees (IACUC) of the University of Illinois at Chicago, Indiana Wesleyan University, Marine Biological Laboratory, and University of California Berkeley as well as the federal guidelines listed in the Public Health Service Policy on Humane Care and Use of Laboratory Animals.

## Author Contributions

RM, BT, JC, PS, RK, and MK all contributed to the writing, content and editing of this article and approved the submitted version.

## Conflict of Interest

The authors declare that the research was conducted in the absence of any commercial or financial relationships that could be construed as a potential conflict of interest.

## Publisher’s Note

All claims expressed in this article are solely those of the authors and do not necessarily represent those of their affiliated organizations, or those of the publisher, the editors and the reviewers. Any product that may be evaluated in this article, or claim that may be made by its manufacturer, is not guaranteed or endorsed by the publisher.

## References

[B1] Abbracchio M. P. Burnstock G. Verkhratsky A. Zimmermann H. (2009). Purinergic signalling in the nervous system: an overview. Trends Neurosci. 32, 19–29. 10.1016/j.tins.2008.10.001 19008000

[B2] Agulhon C. Sun M.-Y. Murphy T. Myers T. Lauderdale K. Fiacco T. A. (2012). Calcium signaling and gliotransmission in normal vs. reactive astrocytes. Front. Pharmacol. 3:139. 10.3389/fphar.2012.00139 22811669 PMC3395812

[B500] Al-Fifi Z. I. Marshall S. L. Hyde D. Anstee J. H. Bowler K. (1998). Characterization of ATPases of apical membrane fractions from Locusta migratoria Malpighian tubules. Insect. Biochem. Mol. Biol. 28, 201–211. 10.1016/s0965-1748(98)00025-3 9684329

[B3] Anderson M. A. Ao Y. Sofroniew M. V. (2014). Heterogeneity of reactive astrocytes. Neurosci. Lett. 565, 23–29. 10.1016/j.neulet.2013.12.030 24361547 PMC3984948

[B4] Ansell B. Clarke E. (1956). Acetazolamide in treatment of epilepsy. Br. Med. J. 1, 650–654. 10.1136/bmj.1.4968.650 20788515 PMC1979321

[B5] Ashhad S. Narayanan R. (2019). Stores, channels, glue, and trees: active glial and active dendritic physiology. Mol. Neurobiol. 56, 2278–2299. 10.1007/s12035-018-1223-5 30014322 PMC6394607

[B6] Baird-Daniel E. Daniel A. G. S. Wenzel M. Li D. Liou J.-Y. Laffont P.. (2017). Glial calcium waves are triggered by seizure activity and not essential for initiating ictal onset or neurovascular coupling. Cereb. Cortex 27, 3318–3330. 10.1152/ajpregu.00048.2021 28369176 PMC6433182

[B7] Bang J. Kim H. Y. Lee H. (2016). Optogenetic and chemogenetic approaches for studying astrocytes and gliotransmitters. Exp. Neurobiol. 25, 205–221. 10.5607/en.2016.25.5.205 27790055 PMC5081467

[B8] Barnes S. (1995). “Photoreceptor synaptic output: neurotransmitter release and photoreceptor coupling,” in Neurobiology and Clinical Aspects of the Outer Retina, eds Djamgoz M. B. Archer S. N. Vallerga S. (Dordrecht: Springer), 133–154.

[B9] Barnes S. Merchant V. Mahmud F. (1993). Modulation of transmission gain by protons at the photoreceptor output synapse. Proc. Natl. Acad. Sci. U S A 90, 10081–10085. 10.1073/pnas.90.21.10081 7694280 PMC47717

[B10] Barres B. A. Freeman M. R. Stevens B. (eds). (2015). Glia. New York, NY: Cold Spring Harbor Laboratory Press.

[B11] Basarsky T. A. Duffy S. N. Andrew R. D. MacVicar B. A. (1998). Imaging spreading depression and associated intracellular calcium waves in brain slices. J. Neurosci. 18, 7189–7199. 10.1523/JNEUROSCI.18-18-07189.1998 9736642 PMC6793239

[B12] Bazargani N. Attwell D. (2016). Astrocyte calcium signaling: the third wave. Nat. Neurosci. 19, 182–189. 10.1038/nn.4201 26814587

[B13] Benemei S. Dussor G. (2019). TRP channels and migraine: recent developments and new therapeutic opportunities. Pharmaceuticals 12:54. 10.3390/ph12020054 30970581 PMC6631099

[B14] Bergstom W. H. Carzoli R. F. Lombroso C. Davidson D. T. Wallace W. M. (1952). Observations on the metabolic and clinical effects of carbonic-anhydrase inhibitors in epileptics. AMA Am. J. Dis. Child 84, 771–772. 12996020

[B16] Binder D. K. Steinhäuser C. (2021). Astrocytes and epilepsy. Neurochem. Res. [Epub ahead of print]. 10.1007/s11064-021-03236-x 33661442

[B17] Bindocci E. Savtchouk I. Liaudet N. Becker D. Carriero G. Volterra A. (2017). Three-dimensional Ca^2+^ imaging advances understanding of astrocyte biology. Science 356:eaai8185. 10.1126/science.aai8185 28522470

[B19] Bjorness T. E. Dale N. Mettlach G. Sonneborn A. Sahin B. Fienberg A. A.. (2016). An adenosine-mediated glial-neuronal circuit for homeostatic sleep. J. Neurosci. 36, 3709–3721. 10.1523/JNEUROSCI.3906-15.2016 27030757 PMC4812131

[B18] Bjorness T. E. Greene R. W. (2009). Adenosine and sleep. Curr. Neuropharmacol. 7, 238–245. 10.2174/157015909789152182 20190965 PMC2769007

[B20] Blutstein T. Haydon P. G. (2013). The importance of astrocyte-derived purines in the modulation of sleep. Glia 61, 129–139. 10.1002/glia.22422 23027687 PMC3527671

[B21] Boer K. Troost D. Jansen F. Nellist M. van den Ouweland A. M. W. Geurts J. J. G.. (2008). Clinicopathological and immunohistochemical findings in an autopsy case of tuberous sclerosis complex. Neuropathology 28, 577–590. 10.1111/j.1440-1789.2008.00920.x 18410267

[B22] Boyman L. Karbowski M. Lederer W. J. (2020). Regulation of mitochondrial ATP production: Ca^2+^ signaling and quality control. Trends Mol. Med. 26, 21–39. 10.1016/j.molmed.2019.10.007 31767352 PMC7921598

[B23] Breton S. Hammar K. Smith P. J. Brown D. (1998). Proton secretion in the male reproductive tract: involvement of Cl—independent HCO^−^_3_ transport. Am. J. Physiol. 275, C1134–C1142. 10.1152/ajpcell.1998.275.4.C1134 9755067

[B24] Burda J. E. Sofroniew M. V. (2014). Reactive gliosis and the multicellular response to CNS damage and disease. Neuron 81, 229–248. 10.1016/j.neuron.2013.12.034 24462092 PMC3984950

[B25] Burkeen J. F. Womac A. D. Earnest D. J. Zoran M. J. (2011). Mitochondrial calcium signaling mediates rhythmic extracellular ATP accumulation in suprachiasmatic nucleus astrocytes. J. Neurosci. 31, 8432–8440. 10.1523/JNEUROSCI.6576-10.2011 21653847 PMC3125703

[B26] Burnstock G. Verkhratsky A. (2012). Purinergic Signalling and the Nervous System. Berlin, Heidelberg: Springer Verlag.

[B27] Cadetti L. Thoreson W. B. (2006). Feedback effects of horizontal cell membrane potential on cone calcium currents studied with simultaneous recordings. J. Neurophysiol. 95, 1992–1995. 10.1152/jn.01042.2005 16371457 PMC2474467

[B28] Cammer W. Tansey F. A. (1988). Carbonic anhydrase immunostaining in astrocytes in the rat cerebral cortex. J. Neurochem. 50, 319–322. 10.1111/j.1471-4159.1988.tb13267.x 2891787

[B29] Cao J. Ribelayga C. P. Mangel S. C. (2020). A circadian clock in the retina regulates rod-cone gap junction coupling and neuronal light responses *via* activation of adenosine A2A receptors. Front. Cell. Neurosci. 14:605067. 10.3389/fncel.2020.605067 33510619 PMC7835330

[B30] Carmignoto G. Haydon P. G. (2012). Astrocyte calcium signaling and epilepsy. Glia 60, 1227–1233. 10.1002/glia.22318 22389222 PMC4532388

[B31] Chang B. S. Lowenstein D. H. (2003). Epilepsy. N. Engl. J. Med. 349, 1257–1266. 10.1056/NEJMra022308 14507951

[B32] Charles A. (2013). Migraine: a brain state. Curr. Opin. Neurol. 26, 235–239. 10.1097/WCO.0b013e32836085f4 23493160

[B33] Charles A. (2018). The migraine aura. Continuum 24, 1009–1022. 10.1212/CON.0000000000000627 30074546

[B34] Charles A. C. Baca S. M. (2013). Cortical spreading depression and migraine. Nat. Rev. Med. 9, 637–644. 10.1038/nrneurol.2013.192 24042483

[B36] Charles A. Brennan K. (2009). Cortical spreading depression-new insights and persistent questions. Cephalalgia 29, 1115–1124. 10.1111/j.1468-2982.2009.01983.x 19735537 PMC5500297

[B40] Chen X. H. Bezprozvanny I. Tsien R. W. (1996). Molecular basis of proton block of L-type Ca^2+^ channels. J. Gen. Physiol. 108, 363–374. 10.1085/jgp.108.5.363 8923262 PMC2229351

[B39] Chen J. C. Chesler M. (1992). pH transients evoked by excitatory synaptic transmission are increased by inhibition of extracellular carbonic anhydrase. Proc. Natl. Acad. Sci. U S A 89, 7786–7790. 10.1073/pnas.89.16.7786 1380165 PMC49796

[B37] Chen H.-Y. Chesler M. (2015). Autocrine boost of NMDAR current in hippocampal CA1 pyramidal neurons by a PMCA-dependent, perisynaptic, extracellular pH shift. J. Neurosci. 35, 873–877. 10.1523/JNEUROSCI.2293-14.2015 25609607 PMC4300330

[B501] Choi J. V. Tchernookova B. K. Kumar W. Kiedrowski L. Goeke C. Guizzetti M.. (2021). Extracellular ATP induced alterations in extracellular H^+^ fluxes from cultured cortical and hippocampal astrocytes. Front Cell Neurosci. 15:640217. 10.3389/fncel.2021.640217 33994945 PMC8120152

[B41] Chuquet J. Hollender L. Nimchinsky E. A. (2007). High-resolution *in vivo* imaging of the neurovascular unit during spreading depression. J. Neurosci. 27, 4036–4044. 10.1523/JNEUROSCI.0721-07.2007 17428981 PMC6672520

[B42] Chu-Shore C. J. Major P. Camposano S. Muzykewicz D. Thiele E. A. (2010). The natural history of epilepsy in tuberous sclerosis complex. Epilepsia 51, 1236–1241. 10.1111/j.1528-1167.2009.02474.x 20041940 PMC3065368

[B43] Çilliler A. E. Güven H. Çomoğlu S. S. (2017). Epilepsy and headaches: further evidence of a link. Epilepsy Behav. 70, 161–165. 10.1016/j.yebeh.2017.03.009 28427026

[B44] Cozzolino O. Marchese M. Trovato F. Pracucci E. Ratto G. M. Buzzi M. G.. (2018). Understanding spreading depression from headache to sudden unexpected death. Front. Neurol. 9:19. 10.3389/fneur.2018.00019 29449828 PMC5799941

[B45] Cui Y. Kataoka Y. Watanabe Y. (2014). Role of cortical spreading depression in the pathophysiology of migraine. Neurosci. Bull. 30, 812–822. 10.1007/s12264-014-1471-y 25260797 PMC5562594

[B46] Curatolo P. Moavero R. de Vries P. J. (2015). Neurological and neuropsychiatric aspects of tuberous sclerosis complex. Lancet Neurol. 14, 733–745. 10.1016/S1474-4422(15)00069-1 26067126

[B47] Curatolo P. Verdecchia M. Bombardieri R. (2002). Tuberous sclerosis complex: a review of neurological aspects. Eur. J. Paediatr. Neurol. 6, 15–23. 10.1053/ejpn.2001.0538 11993952

[B48] Davidoff R. A. (2002). Migraine: Manifestations, Pathogenesis, and Management. 2nd Edn. Oxford: Oxford University Press.

[B49] de Lanerolle N. C. Lee T.-S. Spencer D. D. (2010). Astrocytes and epilepsy. Neurotherapeutics 7, 424–438. 10.1016/j.nurt.2010.08.002 20880506 PMC5084304

[B50] De Simone R. Ranieri A. Marano E. Beneduce L. Ripa P. Bilo L.. (2007). Migraine and epilepsy: clinical and pathophysiological relations. Neurol. Sci. 28, S150–S155. 10.1007/s10072-007-0769-1 17508163

[B51] DeVries S. H. (2001). Exocytosed protons feedback to suppress the Ca^2+^ current in mammalian cone photoreceptors. Neuron 32, 1107–1117. 10.1016/s0896-6273(01)00535-9 11754841

[B52] Dixon S. J. Yu R. Panupinthu N. Wilson J. X. (2004). Activation of P2 nucleotide receptors stimulates acid efflux from astrocytes. Glia 47, 367–376. 10.1002/glia.20048 15293234

[B53] Dmitriev A. V. Mangel S. C. (2000). A circadian clock regulates the pH of the fish retina. J. Physiol. 522, 77–82. 10.1111/j.1469-7793.2000.0077m.x 10618153 PMC2269739

[B54] Dmitriev A. V. Mangel S. C. (2001). Circadian clock regulation of pH in the rabbit retina. J. Neurosci. 21, 2897–2902. 10.1523/JNEUROSCI.21-08-02897.2001 11306641 PMC6762511

[B55] Doering C. J. McRory J. E. (2007). Effects of extracellular pH on neuronal calcium channel activation. Neuroscience 146, 1032–1043. 10.1016/j.neuroscience.2007.02.049 17434266

[B56] Dooves S. van Velthoven A. J. H. Suciati L. G. Heine V. M. (2021). Neuron-glia interactions in tuberous sclerosis complex affect the synaptic balance in 2D and organoid cultures. Cells 10:134. 10.3390/cells10010134 33445520 PMC7826837

[B57] Durkee C. A. Araque A. (2019). Diversity and specificity of astrocyte-neuron communication. Neuroscience 396, 73–78. 10.1016/j.neuroscience.2018.11.010 30458223 PMC6494094

[B58] Dussor G. (2015). ASICs as therapeutic targets for migraine. Neuropharmacol 94, 64–71. 10.1016/j.neuropharm.2014.12.015 25582295 PMC4458434

[B59] Dussor G. (2019). New discoveries in migraine mechanisms and therapeutic targets. Curr. Opin. Physiol. 11, 116–124. 10.1016/j.cophys.2019.10.013 31815209 PMC6897325

[B60] Eikermann-Haerter K. Ayata C. (2010). Cortical spreading depression and migraine. Curr. Neurol. Neurosci. Rep. 10, 167–173. 10.1007/s11910-010-0099-1 20425031

[B61] Eikermann-Haerter K. Negro A. Ayata C. (2013). Spreading depression and the clinical correlates of migraine. Rev. Neurosci. 24, 353–363. 10.1515/revneuro-2013-0005 23907418

[B62] Engel T. Alves M. Sheedy C. Henshall D. C. (2016). ATPergic signalling during seizures and epilepsy. Neuropharmacol 104, 140–153. 10.1016/j.neuropharm.2015.11.001 26549853

[B63] Escartin C. Galea E. Lakatos A. O’Callaghan J. P. Petzold G. C. Serrano-Pozo A.. (2021). Reactive astrocyte nomenclature, definitions and future directions. Nat. Neurosci. 24, 312–325. 10.1038/s41593-020-00783-4 33589835 PMC8007081

[B64] Fellin T. Gomez-Gonzalo M. Gobbo S. Carmignoto G. Haydon P. G. (2006). Astrocytic glutamate is not necessary for the generation of epileptiform neuronal activity in hippocampal slices. J. Neurosci. 26, 9312–9322. 10.1523/JNEUROSCI.2836-06.2006 16957087 PMC6674496

[B65] Fiacco T. A. McCarthy K. D. (2018). Multiple lines of evidence indicate that gliotransmission does not occur under physiological conditions. J. Neurosci. 38, 3–13. 10.1523/JNEUROSCI.0016-17.2017 29298904 PMC5761435

[B66] Fliegel L. (2020). Role of genetic mutations of the Na^+^/H^+^ exchanger isoform 1, in human disease and protein targeting and activity. Mol. Cell. Biochem. 476, 1221–1232. 10.1007/s11010-020-03984-4 33201382

[B67] Gasperini R. J. Pavez M. Thompson A. C. Mitchell C. B. Hardy H. Young K. M.. (2017). How does calcium interact with the cytoskeleton to regulate growth cone motility during axon pathfinding? Mol. Cell. Neurosci. 84, 29–35. 10.1016/j.mcn.2017.07.006 28765051

[B68] Gorji A. (2001). Spreading depression: a review of the clinical relevance. Brain Res. Brain Res. Rev. 38, 33–60. 10.1016/s0165-0173(01)00081-9 11750926

[B70] Grajkowska W. Kotulska K. Jurkiewicz E. Matyja E. (2010). Brain lesions in tuberous sclerosis complex. Review. Folia Neuropathol. 48, 139–149. 20924998

[B71] Greene R. W. Bjorness T. E. Suzuki A. (2017). The adenosine-mediated, neuronal-glial, homeostatic sleep response. Curr. Opin. Neurobiol. 44, 236–242. 10.1016/j.conb.2017.05.015 28633050 PMC5523826

[B72] Gu X. Q. Yao H. Haddad G. G. (2001). Increased neuronal excitability and seizures in the Na^+^/H^+^ exchanger null mutant mouse. Am. J. Physiol. Cell Physiol. 281, C496–C503. 10.1152/ajpcell.2001.281.2.C496 11443048

[B73] Guerra-Gomes S. Sousa N. Pinto L. Oliveira J. F. (2017). Functional roles of astrocyte calcium elevations: from synapses to behavior. Front. Cell. Neurosci. 11:427. 10.3389/fncel.2017.00427 29386997 PMC5776095

[B74] Guthrie P. B. Knappenberger J. Segal M. Bennett M. V. Charles A. C. Kater S. B. (1999). ATP released from astrocytes mediates glial calcium waves. J. Neurosci. 19, 520–528. 10.1523/JNEUROSCI.19-02-00520.1999 9880572 PMC6782195

[B75] Halassa M. M. Fellin T. Haydon P. G. (2007). The tripartite synapse: roles for gliotransmission in health and disease. Trends Mol. Med. 13, 54–63. 10.1016/j.molmed.2006.12.005 17207662

[B76] Halassa M. M. Fellin T. Haydon P. G. (2009). Tripartite synapses: roles for astrocytic purines in the control of synaptic physiology and behavior. Neuropharmacol 57, 343–346. 10.1016/j.neuropharm.2009.06.031 19577581 PMC3190118

[B77] Harriott A. M. Chung D. Y. Uner A. Bozdayi R. O. Morais A. Takizawa T.. (2021). Optogenetic spreading depression elicits trigeminal pain and anxiety behavior. Ann. Neurol. 89, 99–110. 10.1002/ana.25926 33016466 PMC8075185

[B78] Harriott A. M. Takizawa T. Chung D. Y. Chen S.-P. (2019). Spreading depression as a preclinical model of migraine. J. Headache Pain 20:45. 10.1186/s10194-019-1001-4 31046659 PMC6734429

[B79] Harsanyi K. Mangel S. C. (1993). Modulation of cone to horizontal cell transmission by calcium and pH in the fish retina. Vis. Neurosci. 10, 81–91. 10.1017/s0952523800003242 8381021

[B80] Haydon P. G. (2017). Astrocytes and the modulation of sleep. Curr. Opin. Neurobiol. 44, 28–33. 10.1016/j.conb.2017.02.008 28284099 PMC5511068

[B89] Headache Classification Committee of the International Headache Society (IHS). (2013). The international classification of headache disorders, 3rd edition. Cephalalgia 33, 1–211. 10.1177/0333102413485658 29368949

[B81] Henley J. Poo M.-M. (2004). Guiding neuronal growth cones using Ca^2+^ signals. Trends Cell Biol. 14, 320–330. 10.1016/j.tcb.2004.04.006 15183189 PMC3115711

[B82] Heuser D. Astrup J. Lassen N. A. Betz B. E. (1975). Brain carbonic acid acidosis after acetazolamide. Acta Physiol. Scand. 93, 385–390. 10.1111/j.1748-1716.1975.tb05827.x 238362

[B83] Hirasawa H. Kaneko A. (2003). pH changes in the invaginating synaptic cleft mediate feedback from horizontal cells to cone photoreceptors by modulating Ca^2+^ channels. J. Gen. Physiol. 122, 657–671. 10.1085/jgp.200308863 14610018 PMC2229595

[B84] Holland P. R. Akerman S. Andreou A. P. Karsan N. Wemmie J. A. Goadsby P. J. (2012). Acid-sensing ion channel 1: a novel therapeutic target for migraine with aura. Ann. Neurol. 72, 559–563. 10.1002/ana.23653 23109150

[B85] Holst S. C. Landolt H.-P. (2018). Sleep-wake neurochemistry. Sleep Med. Clin. 13, 137–146. 10.1016/j.jsmc.2018.03.002 29759265

[B86] Hoogland T. M. Kuhn B. Göbel W. Huang W. Nakai J. Helmchen F.. (2009). Radially expanding transglial calcium waves in the intact cerebellum. Proc. Natl. Acad. Sci. U S A 106, 3496–3501. 10.1073/pnas.0809269106 19211787 PMC2651231

[B87] Hosoi N. Arai I. Tachibana M. (2005). Group III metabotropic glutamate receptors and exocytosed protons inhibit L-type calcium currents in cones but not in rods. J. Neurosci. 25, 4062–4072. 10.1523/JNEUROSCI.2735-04.2005 15843608 PMC6724956

[B88] Huston J. P. Haas H. L. Boix F. Pfister M. Decking U. Schrader J.. (1996). Extracellular adenosine levels in neostriatum and hippocampus during rest and activity periods of rats. Neuroscience 73, 99–107. 10.1016/0306-4522(96)00021-8 8783234

[B90] Jacoby J. Kreitzer M. A. Alford S. Malchow R. P. (2014). Fluorescent imaging reports an extracellular alkalinization induced by glutamatergic activation of isolated retinal horizontal cells. J. Neurophysiol. 111, 1056–1064. 10.1152/jn.00768.2013 24335210 PMC3949228

[B91] Jacoby J. Kreitzer M. A. Alford S. Qian H. Tchernookova B. K. Naylor E. R.. (2012). Extracellular pH dynamics of retinal horizontal cells examined using electrochemical and fluorometric methods. J. Neurophysiol. 107, 868–879. 10.1152/jn.00878.2011 22090459 PMC3289475

[B92] Jalali-Yazdi F. Chowdhury S. Yoshioka C. Gouaux E. (2018). Mechanisms for zinc and proton inhibition of the GluN1/GluN2A NMDA receptor. Cell 175, 1520.e15–1532.e15. 10.1016/j.cell.2018.10.043 30500536 PMC6333211

[B93] Jha M. K. Morrison B. M. (2018). Glia-neuron energy metabolism in health and diseases: new insights into the role of nervous system metabolic transporters. Exp. Neurol. 309, 23–31. 10.1016/j.expneurol.2018.07.009 30044944 PMC6156776

[B94] Jha M. K. Morrison B. M. (2020). Lactate transporters mediate glia-neuron metabolic crosstalk in homeostasis and disease. Front. Cell. Neurosci. 14:589582. 10.3389/fncel.2020.589582 33132853 PMC7550678

[B95] Kasteleijn-Nolst Trenité D. G. A. Verrotti A. Di Fonzo A. Cantonetti L. Bruschi R. Chiarelli F.. (2010). Headache, epilepsy and photosensitivity: how are they connected? J. Headache Pain 11, 469–476. 10.1007/s10194-010-0229-9 20963464 PMC3476223

[B96] Kater S. B. Mills L. R. (1991). Regulation of growth cone behavior by calcium. J. Neurosci. 11, 891–899. 10.1523/JNEUROSCI.11-04-00891.1991 2010811 PMC6575390

[B97] Keezer M. R. Bauer P. R. Ferrari M. D. Sander J. W. (2015). The comorbid relationship between migraine and epilepsy: a systematic review and meta-analysis. Eur. J. Neurol. 22, 1038–1047. 10.1111/ene.12612 25495495

[B98] Khakh B. S. McCarthy K. D. (2015). Astrocyte calcium signaling: from observations to functions and the challenges therein. Cold Spring Harb. Perspect. Biol. 7:a020404. 10.1101/cshperspect.a020404 25605709 PMC4382738

[B99] Kleinschmidt J. (1991). Signal transmission at the photoreceptor synapse. Role of calcium ions and protons. Ann. N Y Acad. Sci. 635, 468–470. 10.1111/j.1749-6632.1991.tb36529.x 1660251

[B100] Kofuji P. Araque A. (2021). G-protein-coupled receptors in astrocyte-neuron communication. Neuroscience 456, 71–84. 10.1016/j.neuroscience.2020.03.025 32224231 PMC8817509

[B101] Kraig R. P. Nicholson C. (1978). Extracellular ionic variations during spreading depression. Neuroscience 3, 1045–1059. 10.1016/0306-4522(78)90122-7 745780

[B102] Kramer R. H. Davenport C. M. (2015). Lateral inhibition in the vertebrate retina: the case of the missing neurotransmitter. PLoS Biol. 13:e1002322. 10.1371/journal.pbio.1002322 26656622 PMC4675548

[B103] Kreitzer M. A. Collis L. P. Molina A. J. A. Smith P. J. S. Malchow R. P. (2007). Modulation of extracellular proton fluxes from retinal horizontal cells of the catfish by depolarization and glutamate. J. Gen. Physiol. 130, 169–182. 10.1085/jgp.200709737 17664345 PMC2151636

[B104] Kreitzer M. A. Jacoby J. Naylor E. Baker A. Grable T. Tran E.. (2012). Distinctive patterns of alterations in proton efflux from goldfish retinal horizontal cells monitored with self-referencing H^+^-selective electrodes. Eur. J. Neurosci. 36, 3040–3050. 10.1111/j.1460-9568.2012.08226.x 22809323 PMC11342235

[B105] Kreitzer M. A. Swygart D. Osborn M. Skinner B. Heer C. Kaufman R.. (2017). Extracellular H^+^ fluxes from tiger salamander Müller (glial) cells measured using self-referencing H^+^-selective microelectrodes. J. Neurophysiol. 118, 3132–3143. 10.1152/jn.00409.2017 28855292

[B106] Kumaria A. Tolias C. M. Burnstock G. (2008). ATP signalling in epilepsy. Purinergic Signal. 4, 339–346. 10.1007/s11302-008-9115-1 18568425 PMC2583203

[B107] Kunkler P. E. Kraig R. P. (1998). Calcium waves precede electrophysiological changes of spreading depression in hippocampal organ cultures. J. Neurosci. 18, 3416–3425. 10.1523/JNEUROSCI.18-09-03416.1998 9547248 PMC2699599

[B108] Landolt H.-P. Dijk D.-J. (2019). Sleep-Wake Neurobiology and Pharmacology. Cham: Springer International Publishing.

[B109] Lauritzen M. (1994). Pathophysiology of the migraine aura. The spreading depression theory. Brain 117, 199–210. 10.1093/brain/117.1.199 7908596

[B110] Leão A. A. (1944). Spreading depression of activity in the cerebral cortex. J. Neurophysiol. 7, 359–390. 10.1152/jn.1947.10.6.409 20268874

[B111] Liddelow S. A. Barres B. A. (2017). Reactive astrocytes: production, function and therapeutic potential. Immunity 46, 957–967. 10.1016/j.immuni.2017.06.006 28636962

[B112] Losi G. Mariotti L. Sessolo M. Carmignoto G. (2017). New tools to study astrocyte Ca^2+^ signal dynamics in brain networks *in vivo*. Front. Cell. Neurosci. 11:134. 10.3389/fncel.2017.00134 28536505 PMC5422467

[B113] Luppi P.-H. Fort P. (2011). Neurochemistry of sleep an overview of animal experimental work. Handb. Clin. Neurol. 98, 173–190. 10.1016/B978-0-444-52006-7.00011-3 21056186

[B114] Major S. Huo S. Lemale C. L. Siebert E. Milakara D. Woitzik J.. (2020). Direct electrophysiological evidence that spreading depolarization-induced spreading depression is the pathophysiological correlate of the migraine aura and a review of the spreading depolarization continuum of acute neuronal mass injury. Geroscience 42, 57–80. 10.1007/s11357-019-00142-7 31820363 PMC7031471

[B115] Makani S. Chesler M. (2010). Rapid rise of extracellular pH evoked by neural activity is generated by the plasma membrane calcium ATPase. J. Neurophysiol. 103, 667–676. 10.1152/jn.00948.2009 19939954 PMC2822688

[B116] Malarkay E. B. Parpura V. (2011). Temporal characteristics of vesicular fusion in astrocytes: examination of synaptobrevin 2-laden vesicles at single vesicle resolution. J. Physiol. 589, 4271–4300. 10.1113/jphysiol.2011.210435 21746780 PMC3180583

[B117] Malchow R. P. Tchernookova B. K. Holzhausen L. C. Kramer R. H. Kreitzer M. A. (2018). ATP-induced alterations in extracellular H^+^: a potent potential mechanism for modulation of neuronal signals by Müller (glial) cells in the vertebrate retina. Invest Ophthalmol. Visual Sci. 59:1863.

[B118] Mangel S. C. (2001). Circadian clock regulation of neuronal light responses in the vertebrate retina. Prog. Brain Res. 131, 505–518. 10.1016/s0079-6123(01)31040-3 11420966

[B119] Maren T. H. (1988). The kinetics of HCO^−^_3_ synthesis related to fluid secretion, pH control and CO_2_ elimination. Annu. Rev. Physiol. 50, 695–717. 10.1146/annurev.ph.50.030188.003403 3132082

[B120] Marpegan L. Swanstrom A. E. Chung K. Simon T. Haydon P. G. Khan S. K.. (2011). Circadian regulation of ATP release in astrocytes. J. Neurosci. 31, 8342–8350. 10.1523/JNEUROSCI.6537-10.2011 21653839 PMC3135876

[B121] Martins-Ferreira H. do Carmo R. J. (1987). Retinal spreading depression and the extracellular milieu. Can. J. Physiol. Pharmacol. 65, 1092–1098. 10.1139/y87-171 3621034

[B122] Martins-Ferreira H. Nedergaard M. Nicholson C. (2000). Perspectives on spreading depression. Brain Res. Brain Res. Rev. 32, 215–234. 10.1016/s0165-0173(99)00083-1 10751672

[B123] McConnell H. M. Owicki J. C. Parce J. W. Miller D. L. Baxter G. T. Wada H. G.. (1992). The cytosensor microphysiometer: biological applications of silicon technology. Science 257, 1906–1912. 10.1126/science.1329199 1329199

[B124] Menna G. Tong C. K. Chesler M. (2000). Extracellular pH changes and accompanying cation shifts during ouabain-induced spreading depression. J. Neurophysiol. 83, 1338–1345. 10.1152/jn.2000.83.3.1338 10712461

[B126] Messerli M. A. Robinson K. R. Smith P. J. S. (2006). “Electrochemical sensor applications to the study of molecular physiology and analyte flux in plants,” in Plant electrophysiology–Theory and Methods, eds Volkov A. G. (Berlin, Heidelberg: Springer), 73–104.

[B125] Messerli M. A. Smith P. J. S. (2010). Construction, theory and practical considerations for using self-referencing of Ca^2+^-selective microelectrodes for monitoring extracellular Ca^2+^ gradients. Methods Cell Biol. 99, 91–111. 10.1016/B978-0-12-374841-6.00004-9 21035684

[B127] Mishra C. B. Kumari S. Angeli A. Bua S. Mongre R. K. Tiwari M.. (2021). Discovery of potent carbonic anhydrase inhibitors as effective anticonvulsant agents: drug design, synthesis and *in vitro* and *in vivo* investigations. J. Med. Chem. 64, 3100–3114. 10.1021/acs.jmedchem.0c01889 33721499

[B128] Mizuguchi M. Takashima S. (2001). Neuropathology of tuberous sclerosis. Brain Dev. 23, 508–515. 10.1016/s0387-7604(01)00304-7 11701246

[B129] Molina A. J. A. Verzi M. P. Birnbaum A. D. Yamoah E. N. Hammar K. Smith P. J. S.. (2004). Neurotransmitter modulation of extracellular H^+^ fluxes from isolated retinal horizontal cells of the skate. J. Physiol. 560, 639–657. 10.1113/jphysiol.2004.065425 15272044 PMC1665295

[B130] Murillo-Rodriguez E. Blanco-Centurion C. Gerashchenko D. Salin-Pascual R. J. Shiromani P. J. (2004). The diurnal rhythm of adenosine levels in the basal forebrain of young and old rats. Neuroscience 123, 361–370. 10.1016/j.neuroscience.2003.09.015 14698744

[B131] Mutch W. A. Hansen A. J. (1984). Extracellular pH changes during spreading depression and cerebral ischemia: mechanisms of brain pH regulation. J. Cereb. Blood Flow. Metab. 4, 17–27. 10.1038/jcbfm.1984.3 6693512

[B132] Nagelhus E. A. (2005). Carbonic anhydrase XIV is enriched in specific membrane domains of retinal pigment epithelium, Müller cells and astrocytes. Proc. Natl. Acad. Sci. U S A 102, 8030–8035. 10.1073/pnas.0503021102 15901897 PMC1142392

[B133] Newman E. A. (1985). Membrane physiology of retinal glial (Müller) cells. J. Neurosci. 5, 2225–2239. 10.1523/JNEUROSCI.05-08-02225.1985 3874934 PMC6565285

[B134] Newman E. A. (1991). Sodium-bicarbonate cotransport in retinal Müller (glial) cells of the salamander. J. Neurosci. 11, 3972–3983. 10.1523/JNEUROSCI.11-12-03972.1991 1744699 PMC6575291

[B135] Newman E. A. (1994). A physiological measure of carbonic anhydrase in Müller cells. Glia 11, 291–299. 10.1002/glia.440110402 7960033

[B136] Newman E. A. (2001). Propagation of intercellular calcium waves in retinal astrocytes and Müller cells. J. Neurosci. 21, 2215–2223. 10.1523/JNEUROSCI.21-07-02215.2001 11264297 PMC2409971

[B137] Newman E. A. (2003). Glial cell inhibition of neurons by release of ATP. J. Neurosci. 23, 1659–1666. 10.1523/JNEUROSCI.23-05-01659.2003 12629170 PMC2322877

[B138] Newman E. A. Zahs K. R. (1997). Calcium waves in retinal glial cells. Science 275, 844–847. 10.1126/science.275.5301.844 9012354 PMC2410141

[B139] Newman E. A. Zahs K. R. (1998). Modulation of neuronal activity by glial cells in the retina. J. Neurosci. 18, 4022–4028. 10.1523/JNEUROSCI.18-11-04022.1998 9592083 PMC2904245

[B140] Nicholls D. Attwell D. (1990). The release and uptake of excitatory amino acids. Trends Pharmacol. Sci. 11, 462–468. 10.1016/0165-6147(90)90129-v 1980041

[B141] Nicholson C. (1984). Comparative neurophysiology of spreading depression in the cerebellum. An. Acad. Bras. Cienc. 56, 481–494. 6398639

[B142] Nikolic L. Nobili P. Shen W. Audinat E. (2020). Role of astrocyte purinergic signaling in epilepsy. Glia 68, 1677–1691. 10.1002/glia.23747 31705600

[B143] Owe S. G. Marcaggi P. Attwell D. (2006). The ionic stoichiometry of the GLAST glutamate transporter in salamander retinal glia. J. Physiol. 577, 591–599. 10.1113/jphysiol.2006.116830 17008380 PMC1890427

[B144] Pankratov Y. Lalo U. Verkhratsky A. North R. A. (2006). Vesicular release of ATP at central synapses. Pflugers Arch. 452, 589–597. 10.1007/s00424-006-0061-x 16639550

[B145] Papouin T. Dunphy J. Tolman M. Foley J. C. Haydon P. G. (2017). Astrocytic control of synaptic function. Philos. Trans. R. Soc. Lond. B Biol. Sci. 372, 20160154–20160158. 10.1098/rstb.2016.0154 28093548 PMC5247586

[B146] Parisi P. Piccioli M. Villa M. P. Buttinelli C. Kasteleijn-Nolst Trenité D. G. A. (2008). Hypothesis on neurophysiopathological mechanisms linking epilepsy and headache. Med. Hypotheses 70, 1150–1154. 10.1016/j.mehy.2007.11.013 18191908

[B147] Patel D. C. Tewari B. P. Chaunsali L. Sontheimer H. (2019). Neuron-glia interactions in the pathophysiology of epilepsy. Nat. Rev. Neurosci. 20, 282–297. 10.1038/s41583-019-0126-4 30792501 PMC8558781

[B148] Pekny M. Wilhelmsson U. Pekna M. (2014). The dual role of astrocyte activation and reactive gliosis. Neurosci. Lett. 565, 30–38. 10.1016/j.neulet.2013.12.071 24406153

[B149] Peters O. Schipke C. G. Hashimoto Y. Kettenmann H. (2003). Different mechanisms promote astrocyte Ca^2+^ waves and spreading depression in the mouse neocortex. J. Neurosci. 23, 9888–9896. 10.1523/JNEUROSCI.23-30-09888.2003 14586018 PMC6740882

[B150] Petrelli F. Bezzi P. (2016). Novel insights into gliotransmitters. Curr. Opin. Pharmacol. 26, 138–145. 10.1016/j.coph.2015.11.010 26707767

[B151] Pierre K. Pellerin L. (2005). Monocarboxylate transporters in the central nervous system: distribution, regulation and function. J. Neurochem. 94, 1–14. 10.1111/j.1471-4159.2005.03168.x 15953344

[B152] Post R. M. Silberstein S. D. (1994). Shared mechanisms in affective illness, epilepsy and migraine. Neurology 44, S37–47. 7969945

[B153] Ransom B. R. (1992). Glial modulation of neural excitability mediated by extracellular pH: a hypothesis. Prog. Brain Res. 94, 37–46. 10.1016/s0079-6123(08)61737-9 1287724

[B154] Ransom B. R. (2000). Glial modulation of neural excitability mediated by extracellular pH: a hypothesis revisited. Prog. Brain Res. 125, 217–228. 10.1016/S0079-6123(00)25012-7 11098659

[B155] Reiss W. G. Oles K. S. (1996). Acetazolamide in the treatment of seizures. Ann. Pharmacother. 30, 514–519. 10.1177/106002809603000515 8740334

[B156] Ribelayga C. Mangel S. C. (2005). A circadian clock and light/dark adaptation differentially regulate adenosine in the mammalian retina. J. Neurosci. 25, 215–222. 10.1523/JNEUROSCI.3138-04.2005 15634784 PMC6725211

[B157] Ribelayga C. Mangel S. C. (2019). Circadian clock regulation of cone to horizontal cell synaptic transfer in the goldfish retina. PLoS One 14:e0218818. 10.1371/journal.pone.0218818 31461464 PMC6713326

[B158] Rice M. E. Nicholson C. (1988). Behavior of extracellular K^+^ and pH in skate (Raja erinacea) cerebellum. Brain Res. 461, 328–334. 10.1016/0006-8993(88)90263-6 3179721

[B159] Robel S. Sontheimer H. (2016). Glia as drivers of abnormal neuronal activity. Nat. Neurosci. 19, 28–33. 10.1038/nn.4184 26713746 PMC4966160

[B160] Rogawski M. A. (2008). Common pathophysiologic mechanisms in migraine and epilepsy. Arch. Neurol. 65, 709–714. 10.1001/archneur.65.6.709 18541791

[B161] Rose C. R. Felix L. Zeug A. Dietrich D. Reiner A. Henneberger C. (2017). Astroglial glutamate signaling and uptake in the hippocampus. Front. Mol. Neurosci. 10:451. 10.3389/fnmol.2017.00451 29386994 PMC5776105

[B162] Rosenberg S. S. Spitzer N. C. (2011). Calcium signaling in neuronal development. Cold Spring Harb. Perspect. Biol. 3:a004259. 10.1101/cshperspect.a004259 21730044 PMC3179332

[B163] Sahlender D. A. Savtchouk I. Volterra A. (2014). What do we know about gliotransmitter release from astrocytes? Philos. Trans. R. Soc. Lond. B Biol. Sci. 369:20130592. 10.1098/rstb.2013.0592 25225086 PMC4173278

[B164] Savtchouk I. Volterra A. (2018). Gliotransmission: beyond black-and-white. J. Neurosci. 38, 14–25. 10.1523/JNEUROSCI.0017-17.2017 29298905 PMC6705815

[B165] Savtchouk I. Carriero G. Volterra A. (2018). Studying axon-astrocyte functional interactions by 3D two-photon Ca^2+^ imaging: a practical guide to experiments and “big data” analysis. Front. Cell. Neurosci. 12:98. 10.3389/fncel.2018.00098 29706870 PMC5908897

[B166] Schock S. C. Munyao N. Yakubchyk Y. Sabourin L. A. Hakim A. M. Ventureyra E. C. G.. (2007). Cortical spreading depression releases ATP into the extracellular space and purinergic receptor activation contributes to the induction of ischemic tolerance. Brain Res. 1168, 129–138. 10.1016/j.brainres.2007.06.070 17706620

[B167] Semyanov A. Henneberger C. Agarwal A. (2020). Making sense of astrocytic calcium signals-from acquisition to interpretation. Nat. Rev. Neurosci. 19:182. 10.1038/s41583-020-0361-8 32873937

[B168] Shigetomi E. Saito K. Sano F. Koizumi S. (2019). Aberrant calcium signals in reactive astrocytes: a key process in neurological disorders. Int. J. Mol. Sci. 20, 20–996. 10.3390/ijms20040996 30823575 PMC6413203

[B169] Smith P. J. (1995). Non-invasive ion probes–tools for measuring transmembrane ion flux. Nature 378, 645–646. 10.1038/378645a0 8524403

[B170] Smith P. J. S. Collis L. P. Messerli M. A. (2010). Windows to cell function and dysfunction: signatures written in the boundary layers. Bioessays 32, 514–523. 10.1002/bies.200900173 20486138

[B172] Smith P. Hammar K. Porterfield D. M. (1999). Self-referencing, non-invasive, ion selective electrode for single cell detection of trans-plasma membrane calcium flux. Microsc. Res. Tech. 46, 398–417. 10.1002/(SICI)1097-0029(19990915)46:6<398::AID-JEMT8>3.0.CO;2-H 10504217

[B173] Smith P. Sanger R. H. Messerli M. A. (2007). “Principles, development and applications of self-referencing electrochemical microelectrodes to the determination of fluxes at cell membranes,” in Electrochemical Methods for Neuroscience, eds Michael A. C. Borland L. M. (Boca Raton, FL: Electrochemical Methods for Neuroscience), 373–405. 21204387

[B171] Smith P. Trimarchi J. (2001). Noninvasive measurement of hydrogen and potassium ion flux from single cells and epithelial structures. Am. J. Physiol., Cell Physiol. 280, C1–C11. 10.1152/ajpcell.2001.280.1.C1 11121371

[B174] Sofroniew M. V. Vinters H. V. (2010). Astrocytes: biology and pathology. Acta Neuropathol. 119, 7–35. 10.1007/s00401-009-0619-8 20012068 PMC2799634

[B175] Somieski P. Nagel W. (2001). Measurement of pH gradients using an ion-sensitive vibrating probe technique (IP). Pflugers Arch. 442, 142–149. 10.1007/s004240000505 11374062

[B176] Somjen G. G. (2005). Aristides Leão’s discovery of cortical spreading depression. J. Neurophysiol. 94, 2–4. 10.1152/classicessays.00031.2005 15985690

[B177] Stafstrom C. E. Carmant L. (2015). Seizures and epilepsy: an overview for neuroscientists. Cold Spring Harb. Perspect. Med. 5:a022426. 10.1101/cshperspect.a022426 26033084 PMC4448698

[B178] Syková E. Nicholson C. (2008). Diffusion in brain extracellular space. Physiol. Rev. 88, 1277–1340. 10.1152/physrev.00027.2007 18923183 PMC2785730

[B179] Takizawa T. Ayata C. Chen S.-P. (2020). Therapeutic implications of cortical spreading depression models in migraine. Prog. Brain Res. 255, 29–67. 10.1016/bs.pbr.2020.05.009 33008510

[B180] Tchernookova B. K. Gongwer M. W. George A. Goeglein B. Powell A. M. Caringal H. L.. (2021). ATP-mediated increase in H^+^ flux from retinal Müller cells: a role for Na^+^/H^+^ exchange. J. Neurophysiol. 125, 184–198. 10.1152/jn.00546.2020 33206577

[B181] Tchernookova B. K. Heer C. Young M. Swygart D. Kaufman R. Gongwer M.. (2018). Activation of retinal glial (Müller) cells by extracellular ATP induces pronounced increases in extracellular H^+^ flux. PLoS One 13:e0190893. 10.1371/journal.pone.0190893 29466379 PMC5821311

[B182] Teive H. A. G. Kowacs P. A. Maranhão Filho P. Piovesan E. J. Werneck L. C. (2005). Leao’s cortical spreading depression: from experimental ‘artifact’ to physiological principle. Neurology 65, 1455–1459. 10.1212/01.wnl.0000183281.12779.cd 16275835

[B183] Theparambil S. M. Hosford P. S. Ruminot I. Kopach O. Reynolds J. R. Sandoval P. Y.. (2020). Astrocytes regulate brain extracellular pH *via* a neuronal activity-dependent bicarbonate shuttle. Nat. Commun. 11:5073. 10.1038/s41467-020-18756-3 33033238 PMC7545092

[B184] Theparambil S. M. Naoshin Z. Defren S. Schmaelzle J. Weber T. Schneider H.-P.. (2017). Bicarbonate sensing in mouse cortical astrocytes during extracellular acid/base disturbances. J. Physiol. 595, 2569–2585. 10.1113/JP273394 27981578 PMC5390880

[B185] Theparambil S. M. Ruminot I. Schneider H. P. Shull G. E. Deitmer J. W. (2014). The electrogenic sodium bicarbonate cotransporter NBCe1 is a high-affinity bicarbonate carrier in cortical astrocytes. J. Neurosci. 34, 1148–1157. 10.1523/JNEUROSCI.2377-13.2014 24453308 PMC6705302

[B186] Thiry A. Dogné J.-M. Supuran C. T. Masereel B. (2007). Carbonic anhydrase inhibitors as anticonvulsant agents. Curr. Top Med. Chem. 7, 855–864. 10.2174/156802607780636726 17504130

[B187] Thomas R. C. (2009). The plasma membrane calcium ATPase (PMCA) of neurones is electroneutral and exchanges 2 H^+^ for each Ca^2+^ or Ba^2+^ ion extruded. J. Physiol. 587, 315–327. 10.1113/jphysiol.2008.162453 19064619 PMC2670047

[B188] Thoreson W. B. Mangel S. C. (2012). Lateral interactions in the outer retina. Prog. Retin. Eye Res. 31, 407–441. 10.1016/j.preteyeres.2012.04.003 22580106 PMC3401171

[B189] Tian G.-F. Azmi H. Takano T. Xu Q. Peng W. Lin J.. (2005). An astrocytic basis of epilepsy. Nat. Med. 11, 973–981. 10.1038/nm1277 16116433 PMC1850946

[B191] Tong C. K. Brion L. P. Suarez C. Chesler M. (2000). Interstitial carbonic anhydrase (CA) activity in brain is attributable to membrane-bound CA type IV. J. Neurosci. 20, 8247–8253. 10.1523/JNEUROSCI.20-22-08247.2000 11069930 PMC6773166

[B190] Tong C. K. Chesler M. (2000). Modulation of spreading depression by changes in extracellular pH. J. Neurophysiol. 84, 2449–2457. 10.1152/jn.2000.84.5.2449 11067987

[B192] Toth A. B. Hori K. Novakovic M. M. Bernstein N. G. Lambot L. Prakriya M. (2019). CRAC channels regulate astrocyte Ca^2+^ signaling and gliotransmitter release to modulate hippocampal GABAergic transmission. Sci. Signal 12:eaaw5450. 10.1126/scisignal.aaw5450 31113852 PMC6837172

[B193] Traynelis S. F. Cull-Candy S. G. (1990). Proton inhibition of N-methyl-D-aspartate receptors in cerebellar neurons. Nature 345, 347–350. 10.1038/345347a0 1692970

[B194] Traynelis S. F. Hartley M. Heinemann S. F. (1995). Control of proton sensitivity of the NMDA receptor by RNA splicing and polyamines. Science 268, 873–876. 10.1126/science.7754371 7754371

[B195] Traynelis S. F. Wollmuth L. P. McBain C. J. Menniti F. S. Vance K. M. Ogden K. K.. (2010). Glutamate receptor ion channels: structure, regulation and function. Pharmacol. Rev. 62, 405–496. 10.1124/pr.109.002451 20716669 PMC2964903

[B196] Vandenberg R. J. Ryan R. M. (2013). Mechanisms of glutamate transport. Physiol. Rev. 93, 1621–1657. 10.1152/physrev.00007.2013 24137018

[B197] Vargas-Sánchez K. Mogilevskaya M. Rodríguez-Pérez J. Rubiano M. G. Javela J. J. González-Reyes R. E. (2018). Astroglial role in the pathophysiology of status epilepticus: an overview. Oncotarget 9, 26954–26976. 10.18632/oncotarget.25485 29928494 PMC6003549

[B198] Verkhratsky A. (2019). Astroglial calcium signaling in aging and Alzheimer’s disease. Cold Spring Harb. Perspect. Biol. 11:a035188. 10.1101/cshperspect.a035188 31110130 PMC6601464

[B15] von Bernhardi R. (ed.). (2016). Glial Cells in Health and Disease of the CNS. Cham: Springer International Publishing.

[B199] Wang T.-M. Holzhausen L. C. Kramer R. H. (2014). Imaging an optogenetic pH sensor reveals that protons mediate lateral inhibition in the retina. Nat. Neurosci. 17, 262–268. 10.1038/nn.3627 24441679 PMC3985427

[B200] Weissman T. A. Riquelme P. A. Ivic L. Flint A. C. Kriegstein A. R. (2004). Calcium waves propagate through radial glial cells and modulate proliferation in the developing neocortex. Neuron 43, 647–661. 10.1016/j.neuron.2004.08.015 15339647

[B201] Womac A. D. Burkeen J. F. Neuendorff N. Earnest D. J. Zoran M. J. (2009). Circadian rhythms of extracellular ATP accumulation in suprachiasmatic nucleus cells and cultured astrocytes. Eur. J. Neurosci. 30, 869–876. 10.1111/j.1460-9568.2009.06874.x 19712092 PMC2757148

[B202] Wu S. M. (1994). Synaptic transmission in the outer retina. Annu. Rev. Physiol. 56, 141–168. 10.1146/annurev.ph.56.030194.001041 8010738

[B203] Xie A. X. Petravicz J. McCarthy K. D. (2015). Molecular approaches for manipulating astrocytic signaling *in vivo*. Front. Cell. Neurosci. 9:144. 10.3389/fncel.2015.00144 25941472 PMC4403552

[B204] Yu X. Nagai J. Khakh B. S. (2020). Improved tools to study astrocytes. Nat. Rev. Neurosci. 21, 121–138. 10.1038/s41583-020-0264-8 32042146

[B205] Zanotti S. Charles A. (1997). Extracellular calcium sensing by glial cells: low extracellular calcium induces intracellular calcium release and intercellular signaling. J. Neurochem. 69, 594–602. 10.1046/j.1471-4159.1997.69020594.x 9231716

[B206] Zarcone D. Corbetta S. (2017). Shared mechanisms of epilepsy, migraine and affective disorders. Neurol. Sci. 38, 73–76. 10.1007/s10072-017-2902-0 28527083

[B207] Zerangue N. Kavanaugh M. P. (1996). Flux coupling in a neuronal glutamate transporter. Nature 383, 634–637. 10.1038/383634a0 8857541

[B208] Zhao H. Carney K. E. Falgoust L. Pan J. W. Sun D. Zhang Z. (2016). Emerging roles of Na^+^/H^+^ exchangers in epilepsy and developmental brain disorders. Prog. Neurobiol. 138, 19–35. 10.1016/j.pneurobio.2016.02.002 26965387 PMC4852136

[B209] Zimmer T. S. Broekaart D. W. M. Gruber V.-E. van Vliet E. A. Mühlebner A. Aronica E. (2020). Tuberous sclerosis complex as disease model for investigating mTOR-related gliopathy during epileptogenesis. Front. Neurol. 11:1028. 10.3389/fneur.2020.01028 33041976 PMC7527496

